# Determining the cortical, corticospinal, and reticulospinal responses to metronome-paced and self-paced strength training

**DOI:** 10.1007/s00421-025-05939-3

**Published:** 2025-08-31

**Authors:** Yonas Akalu, Jamie Tallent, Ashlyn K. Frazer, Ummatul Siddique, Mohamad Rostami, Glyn Howatson, Simon Walker, Dawson J. Kidgell

**Affiliations:** 1 Department of Physiotherapy, School of Primary and Allied Health Care, Faculty of Medicine, Nursing and Health Science, Monash University Exercise Neuroplasticity Research Unit, Monash University, PO Box 527, Frankston, Melbourne, VIC 3199 Australia; 2 Department of Human Physiology, School of Medicine, University of Gondar, Gondar, Ethiopia; 3 School of Sport, Rehabilitation and Exercise Sciences, University of Essex, Colchester, UK; 4 Department of Sport, Exercise and Rehabilitation, Faculty of Health and Life Sciences, Northumbria University, Newcastle-Upon-Tyne, UK; 5 Water Research Group, North West University, Potchefstroom, South Africa; 6 NeuroMuscular Research Center, Faculty of Sport and Health Sciences, University of Jyväskylä, Jyväskylä, Finland

**Keywords:** Resistance training, Rate of force development, Reticulospinal tract, ICAR, TMS

## Abstract

**Purpose:**

The acute neurophysiological responses to resistance training (RT), particularly in corticospinal and reticulospinal pathways, remain unclear. This study investigated the effects of different RT modalities on these pathways.

**Methods:**

Thirty-six RT-naive participants (10 males, 2 females per group) were randomly assigned to metronome-paced RT (MP-RT), self-paced RT (SP-RT), or a control group. Cortical, corticospinal, and cortico-reticulospinal responses were assessed using transcranial magnetic stimulation (TMS), while reticulospinal tract (RST) excitability was evaluated by examining the effect of startle stimulus on rate of force development (RFD) at baseline, 5 min, and 30 min post-exercise.

**Results:**

MP-RT enhanced corticospinal excitability by 50% at 5 min (*p* = 0.017) and 72% at 30 min (*p* < 0.001). MP-RT reduced short-interval cortical inhibition (SICI) by 56% and cSP by 12% at 5 min and ~ 20% at 30 min. SP-RT reduced cSP by 17% at 5 min at 150% active motor threshold (AMT; *p* < 0.05). At 170% AMT, cSP reductions were observed in both MP-RT (23%) and SP-RT (18.9%; *p* < 0.001). SP-RT increased ipsilateral to contralateral motor evoked potential amplitude ratio (ICAR) by 48% at 30 min (*p* < 0.001), and RFD during the initial 50 ms under startling stimuli by 60% at 30 min (*p* = 0.039).

**Conclusion:**

MP-RT enhances intracortical/corticospinal excitability and may support rehabilitation from corticospinal injury/impairment, while SP-RT improves cortico-reticular and reticulospinal excitability, making it suitable for athletes or older adults seeking improved gross strength.

**Graphical Abstract:**

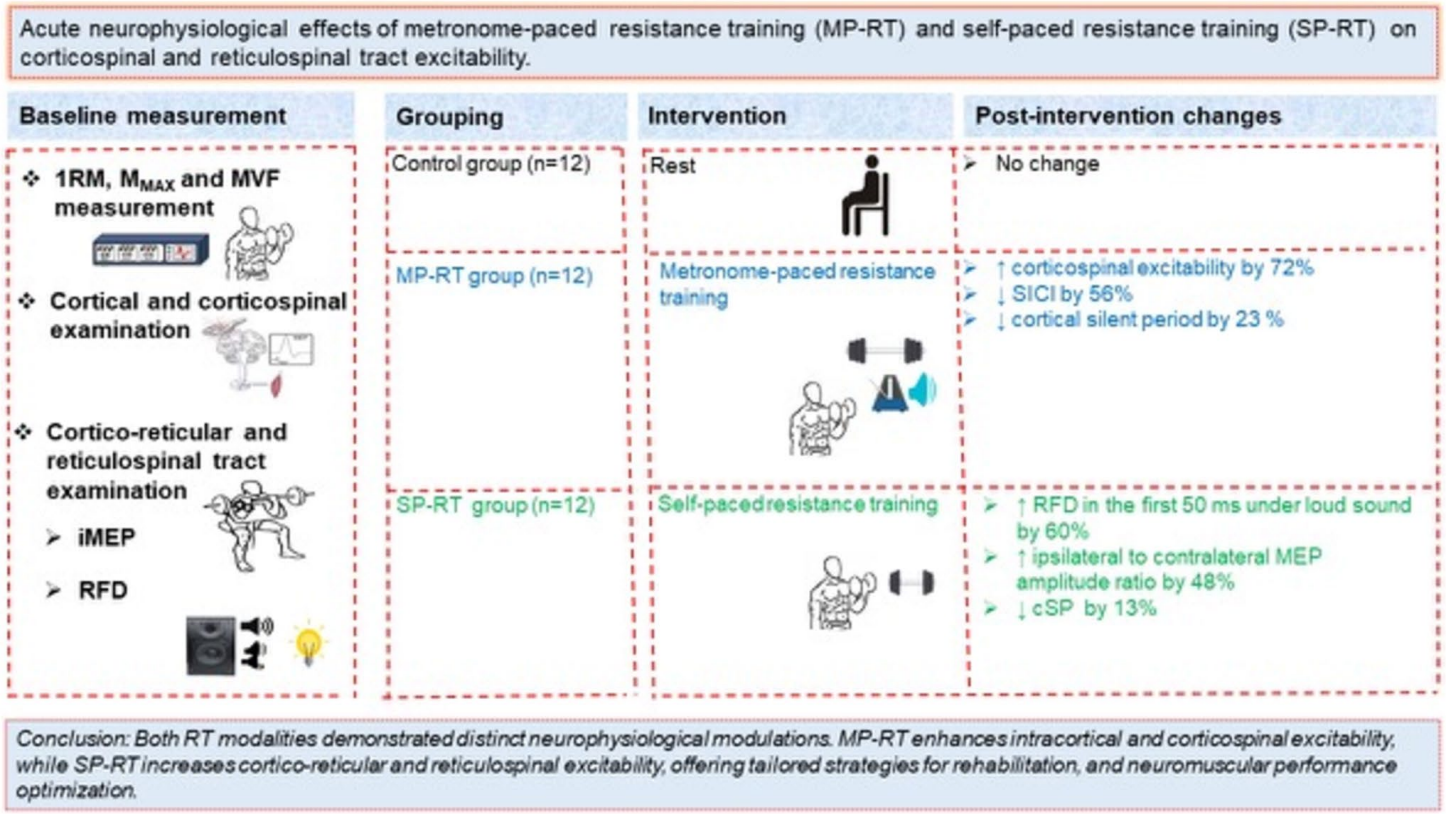

**Supplementary Information:**

The online version contains supplementary material available at 10.1007/s00421-025-05939-3.

## Introduction

Resistance training (RT) is a specialized form of motor training that significantly enhances muscle strength and provides a multitude of physiological benefits across various populations, including athletes and patients (Škarabot et al 2021). Within the domains of athletic training, rehabilitation, and general RT practices, diverse methodologies are employed to achieve targeted improvements in muscle strength (American College of Sports Medicine, 2009). Engaging in RT, particularly with training at a high percentage of maximum or towards failure, augments the force-generating capacity of the involved muscles (Carroll 2012). The initial increase in muscle strength observed following RT is predominantly due to neural adaptations (Siddique et al 2020b; Škarabot et al 2021). However, the exact locus of these neural adaptations remains uncertain (Škarabot et al 2021). To address this uncertainty, the present study investigated the acute effects of RT, specifically examining how metronome-paced (MP) and self-paced (SP) protocols influence corticospinal and cortico-reticulospinal excitability. We hypothesized that these distinct RT modalities would differentially modulate the excitability of these neural pathways.

Several studies have indicated that neural adaptations may begin following a single session of RT (Latella et al 2017; Mason et al 2019a; Muellbacher et al 2001). However, the results from these studies are inconsistent. Some research has reported increased corticospinal excitability (CSE) (Selvanayagam et al 2011) and decreased short-interval intracortical inhibition (SICI) (Hendy & Kidgell 2014; Latella et al 2016; Leung et al 2015) following a single session of heavy load RT. A meta-analysis by Mason et al. (2019b) summated that a single bout of RT decreased the cortical silent period (cSP), increased CSE and intracortical facilitation (ICF), but had no effect on SICI (Mason et al 2019b). One possible reason for these inconsistent findings could be the effect of RT modality or type. There are two common types of RT based on the pacing or timing of the repetitions performed: MP-RT and SP-RT. MP-RT involves performing both concentric and eccentric movements in a timed sequence with each segment’s duration controlled externally by a metronome (Siddique et al 2020a). In contrast, SP-RT involves performing repetitions at an unstructured and typically undulating rhythm without predetermined timing for the concentric or eccentric phases.

This difference in RT pacing has been shown to affect acute cortical and corticospinal tract changes differently (Leung et al 2017; Siddique et al 2020a). For instance, MP-RT increased CSE and decreased SICI, while SP-RT resulted in no changes in CSE or SICI (Leung et al 2015; 2017). Importantly, strength gains occur irrespective of whether RT is paced or not (Leung et al 2017). This finding implies that other neural components within the central nervous system (CNS), such as the reticulospinal tract (RST), may play a role in the strength gains observed during SP-RT.

The RST is a bilateral descending pathway originating in the pontine and medullary regions of the reticular formation. It has the highest density of projections to axial and proximal muscles (Stokes 2004) and is involved in gross motor functions (Akalu et al 2023). On the other hand, the corticospinal tract (CST) is most prominent in the smaller distal limb muscles, and it facilitates fine motor control through its diverse connections (Jang 2014; Zaaimi et al 2018). The anatomical structure of the RST is optimized for executing forceful movements, as it possesses highly divergent postsynaptic connections that allow it to innervate numerous motor units (Atkinson et al 2022; Baker and Perez 2017). A study on non-human primates showed that RST underpinned the increased force production following RT (Glover and Baker 2020), possibly through corticoreticular connections (Škarabot et al 2021). Despite the significant interest in the RST, the effects of RT on its excitability in healthy individuals remain unexplored and underscore the need for further investigation into the RST’s contribution to strength adaptations in humans. This research gap likely stems from the methodological challenges inherent in assessing the RST due to its deep location within the brainstem.

Non-invasive techniques, such as transcranial magnetic stimulation (TMS), which are used to evaluate cortical and corticospinal excitability, are ineffective in directly stimulating RST to examine its response to RT. Nevertheless, other methods such as the StartReact protocol and the measurement of ipsilateral motor evoked potentials (iMEP) elicited by TMS with strong background contraction (Wassermann et al 1991, 1994) (Hu et al 2024) are now being used to investigate RST excitability (Škarabot et al 2022; Tazoe and Perez 2014; Walker et al 2024; Wassermann et al 1991). Transcranial magnetic stimulation over the primary motor cortex (M1) can indirectly activate reticulospinal cells through cortico-reticular connections (Fisher et al 2012). The RST then projects bilaterally to the spinal cord, and therefore, an ipsilateral response (iMEP) can be obtained even by stimulating one cortical hemisphere, suggesting the role of iMEP in assessing RST excitability (Fisher et al 2012). With these methodological advancements, including iMEP and the StartReact protocol, there has been a growing interest in exploring the role of the RST in strength gain (Akalu et al 2023). Using these techniques, two recent cross-sectional studies have demonstrated that individuals with a high level of muscular strength, who were already engaged in RT, exhibited greater excitability of cortico-reticular (Akalu et al 2024) and RST (Akalu et al 2024; Colomer-Poveda et al 2023a) compared to the non-trained group. However, it is important to note that these studies were cross-sectional in nature and therefore cannot establish a cause-effect relationship. Consequently, there is a need for an interventional study to further investigate the effects of RT on both cortico-reticular and RST. Therefore, the present study, for the first time in humans, examined the acute cortico-reticular and reticulospinal responses following a single session (4 sets) of RT. Moreover, we tested whether the different types of RT (MP-RT versus SP-RT) differentially modulate cortical, corticospinal, cortico-reticular, and RST excitability. This line of inquiry is grounded in previous research in primates (Glover and Baker 2020), which indicates that RT is associated with neural adaptations primarily in intracortical and reticulospinal circuits, rather than corticospinal pathways. Therefore, we hypothesized that MP-RT would result in increased intracortical and corticospinal excitability, while SP-RT would result in increased excitability of the cortico-reticular and RST. Understanding these acute changes could clarify the neural mechanisms underlying long-term training adaptations and pinpoint specific sites within the CNS that undergo adaptation.

## Materials and methods

### Subjects

A total of 36 healthy adults (27 males, 9 females; mean age 28.5 ± 9 years), naive to RT, volunteered to participate in this study. The sample size for this study was calculated using G*Power software (version 3.1.7.9). The parameters included a statistical power (β) of 0.80, a significance level (α) of 0.05, and an effect size (Cohen’s f) of 0.25. This effect size was derived from Cohen’s d (0.35) for the shortening of the cSP following short-term RT, as reported by Kidgell et al. (Kidgell and Pearce 2010). Accordingly, the minimum sample size was determined to be 30.

Prior to participation, all subjects provided written informed consent. Subjects were meticulously screened to ensure eligibility for TMS testing and high-intensity RT. All participants had no history of musculoskeletal diseases or injuries and were screened using a TMS adult safety screening questionnaire to exclude those with potential contraindications, such as cranial implants (e.g., metal in the body), a history of head trauma, concussion, seizures, epilepsy, use of medications affecting synaptic plasticity (e.g., antidepressants), or any pre-existing neurological disorders (Rossi et al 2009).

To avoid the effects of circadian rhythm, testing was conducted at the same time of day for all participants, primarily between 12:00 pm and 6:00 pm (Douglas et al 2021). Participants were instructed to avoid unfamiliar strenuous physical activity for 36 h prior to the testing session and to refrain from consuming caffeinated and alcoholic beverages on the day of the session to prevent confounders to TMS responses. Additionally, female participants were advised to ensure they were not in the menstruation phase during the two sessions. Participants were allocated to the three groups using a stratified randomization method to ensure balance in age, sex, and baseline strength across the groups: MP-RT (age: 28 ± 6.6 years), SP-RT (age: 29 ± 4.6 years), or a control group (age: 27 ± 6.8 years), with 12 participants in each group (9 males and 3 females per group). Stratification was based on baseline strength, sex, and age to minimize potential confounding effects. Participants were first categorized into strata according to these variables. Within each stratum, participants were then randomly assigned to one of the three groups using a computer-generated randomization sequence, ensuring an approximately equal distribution across groups. This method ensured balanced allocation, reducing the risk of systematic bias in group comparisons. Additionally, efforts were made to ensure no significant differences in handedness between groups, further reducing potential bias in motor performance and neurophysiological responses. Handedness was assessed using the Edinburgh Handedness Inventory, confirming that all participants were right-handed, with ranges from a minimum of 50 to a maximum of 100 (Oldfield 1971). No adverse effects were reported by any participants during or after the study.

### Experimental setup

Participants attended the laboratory for two sessions: one for familiarization and the other for testing. During the familiarization session, their elbow flexor muscle strength in the dominant hand was evaluated using the one-repetition maximum (1-RM) and maximal voluntary isometric contraction (MVIC). Subsequently, participants were randomly allocated to one of three groups: MP-RT, SP-RT, or a control group. Additionally, participants were acquainted with the basic experimental procedures of the study, which included familiarization with the rate of force development (RFD) and iMEP measurement, exposure to TMS and surface electromyography (sEMG), and conducting peripheral nerve electrical stimulation. Detailed instructions on how to perform RT by synchronizing their movements with a metronome were provided specifically for the MP-RT group.

**Fig. 1:**
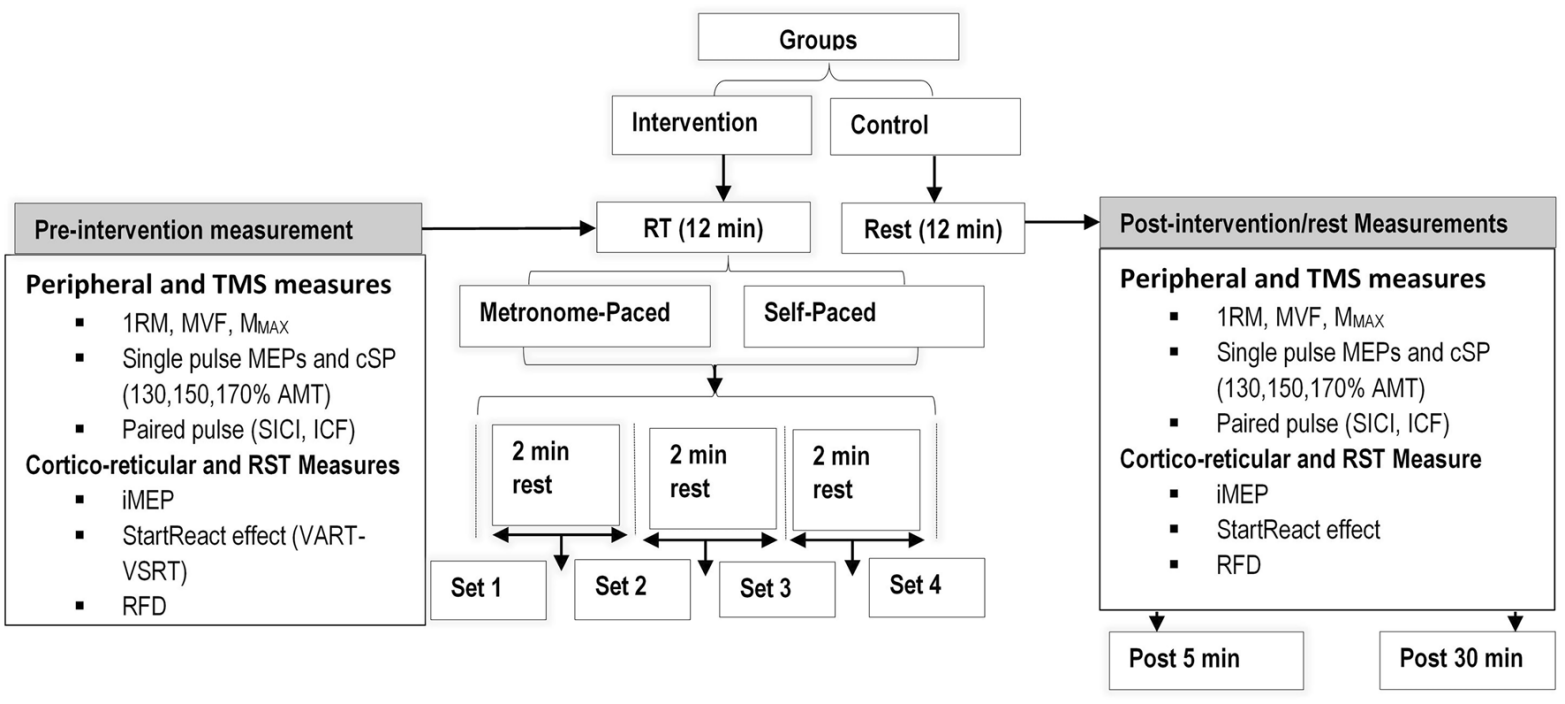
Schematic representation of the experimental design. Measurement of cortical, corticospinal, corticoreticular, and reticulospinal changes following a single session RT. The resistance training involved metronome-paced and self-paced RT. *1RM* one repetition maximum, *AMT* active motor threshold, *cSP* cortical silent period, *ICAR* ipsilateral to contralateral motor evoked potential amplitude ratio, *ICF* intracortical facilitation, *M*_*MAX*_ maximum M-wave, *MVF* maximum voluntary force, *MEPs* motor evoked potentials, *RT* resistance training, *RST* reticulospinal tract, *RFD* rate of force development, *SICI* short interval cortical inhibition

Participants then visited the laboratory for the testing session after a one-week washout period following the familiarization session. During the testing session, baseline (pre-intervention) and post-intervention cortical, corticospinal, cortico-reticulospinal, and RST responses were examined. The intervention involved a single session of RT with 4 sets of 6–8 repetitions of biceps curls for the SP-RT and MP-RT groups, while the control group rested for an equivalent duration. As neural excitability changes reach measurable peaks at various time points post-exercise, post-intervention measurements were conducted after a 5-min rest period following the RT session to minimise the impact of immediate muscle effects such as post-activation facilitation of MEPs and muscle twitch fatigue (Lentz & Nielsen 2002; Muellbacher et al 2002). The second measurement was obtained 30 min following intervention completion, aligning with established post-exercise monitoring protocols. This timing is supported by empirical evidence demonstrating that neurophysiological adaptations evolve temporally and can persist for up to 30 min post-exercise, with the duration modulated by both intervention intensity and modality (Hendy et al 2022; Samii et al 1996). The standardized 30-min interval serves a dual purpose: it enables adequate consolidation of neural adaptations while mitigating the influence of confounding acute physiological responses, including exercise-induced fatigue and stress-mediated autonomic changes. The post-intervention assessment protocol required approximately 20 min at each time point, while baseline measurements necessitated a minimum duration of 40 min to ensure comprehensive data collection (Fig. 1).

### Surface electromyography

Participants were seated in an upright position with a hip joint angle of 90°, using an adjustable chair. Their right elbow, forearm, and hand (dominant hand) were placed in a supinated position on an arm bar, with their shoulders relaxed and their elbow flexed at 90°. The forearm was aligned with the fulcrum of the force transducer and secured against a padded restraint to maintain a supinated hand position under the force transducer (Futek Force Transducer LSB302, Melbourne). Surface electromyographic recordings were taken from the biceps brachii muscle of the dominant arm at baseline, and then again at 5 and 30 min after a single resistance exercise session (rest for the control group). Bipolar surface electrodes (Ag–AgCl) were positioned on the muscle belly, 2 cm apart from each other. They were placed one-third of the way from the antecubital fossa (the crease of the elbow joint), which served as the reference point, to the acromion process (Siddique et al 2024). The electrodes were aligned along the line that connects the medial acromion to the antecubital fossa. Bipolar surface electrodes (Ag–AgCl) were also placed on the biceps brachii of the non-dominant arm for the measurement of the iMEP. To minimize interference and enhance signal quality, the grounding strap was placed around the wrist of the left arm. We adhered to the European recommendations for sEMG as outlined by SENIAM for the non-invasive assessment of muscle, specifically regarding surface electrode placement (Hermens et al 1999). Before placing the electrodes, the skin was cleansed with 70% isopropyl alcohol and lightly abraded to reduce skin impedance, thereby enhancing the transmission of sEMG signals from the muscle fibers to the electrodes (Gilmore and Meyers 1983). Then the surface electrodes were affixed to the skin using adhesive tape. The sEMG signal was amplified (1000 times), bandpass filtered (high pass at 13 Hz, low pass at 1000 Hz), digitized in real-time at a rate of 20 kHz, and recorded and analyzed using PowerLab 4/26 (AD Instruments, Bella Vista, Australia). Both sEMG and force visual feedback were displayed on a computer monitor placed 1 m away from the participant's head, at eye level.

### Strength measurements

Both 1RM and MVIC were measured to assess dynamic and isometric strength of the biceps brachii in the dominant arm, respectively. For the single RT session, a load of 70 to 75% of 1RM was utilized (Leung et al 2017; Siddique et al 2024), which aligns with the recommended training load for novice individuals aiming to maximize muscle strength (Medicine, 2009). The initial weight for the 1RM test was estimated based on the participants’ self-assessment of their strength. During the 1RM test, all participants stood with their backs against the wall to prevent any unnecessary body movements. They held a dumbbell in one hand, with the elbow fully extended and the forearm supinated. Participants then flexed their elbow to lift the dumbbell, receiving verbal encouragement to exert maximum effort. If the participant successfully completed the trial, the weight of the dumbbell was increased by 0.25–0.5 kg. After a 3-min recovery period to minimize muscular fatigue, the process was repeated (Kidgell et al 2010). This progression continued until participants could no longer perform the exercise (biceps curl) through a full range of motion, and the highest weight lifted through a full range of motion was recorded as the 1RM (Jensen et al 2005). This procedure was shown to be highly reliable (ICC = 0.980) (Kidgell et al 2010). To measure MVIC force of the biceps brachii in the dominant arm, participants sat in a chair with relaxed shoulders and their elbows flexed at 90^0^. They were instructed to keep their hands in a supinated position under the force transducer (Futek Force Transducer LSB302, Melbourne) fixed on a height-adjustable table. Participants were instructed to exert maximum force on the transducer for three seconds, with verbal encouragement provided during each attempt/contraction. Simultaneously, real-time visual feedback of the force levels was provided to the participants through a computer monitor positioned one meter in front of them. Two consecutive trials were done, with a three-minute interval between each to mitigate fatigue. The highest force achieved during these trials was recorded as the MVIC. However, if the discrepancy between the results of the initial two trials exceeded 5%, a third trial was administered. Once MVIC was established, 10% was used as a target for subsequent neurophysiological measurements.

### Resistance training protocol

After baseline measurements, participants in the intervention groups (MP-RT and SP-RT) underwent a single session of heavy load unilateral RT using their dominant arm. For the MP-RT group, participants completed four sets of 6–8 repetitions of a standard biceps curl at 70–75% of their 1-RM, as recommended by the ACSM (2009). Participants synchronized their biceps curls with a metronome beat, ensuring consistent timing throughout both the eccentric and concentric phases of the movement. The concentric (i.e., lifting) phase lasted for three seconds, while the eccentric (lowering) phase lasted for four seconds. A two-minute rest period was provided between each set to allow for recovery. Continuous verbal encouragement was given to ensure maximum effort during each set. Participants in the SP-RT group performed the same training, but determined the timing of repetitions themselves, rather than using a metronome. In contrast, participants in the control group rested for 10–12 min, matching the duration of the intervention period for the training groups.

### Maximum compound action potential

For the normalization of TMS responses**,** we elicited the maximal compound action potential (M-wave) from the right brachial plexus at Erb’s point using electrical stimulation with the DS7A Bipolar constant current stimulator (DS7A, Digitimer, Hertfordshire, UK) at a pulse width of 200 μs. The stimulating electrodes (3.2 cm in diameter, Axelgaard Manufacturing Co., LTD) were positioned with the cathode located in the supraclavicular fossa (Erb’s point), while the anode was placed on the acromion. Stimulation was initiated to elicit a direct response from the biceps brachii, starting with low-intensity stimuli while the participant maintained a low-level isometric contraction at 10% of their maximum voluntary isometric contraction (MVIC). This approach ensured consistent and reliable M-wave measurements, as conduction velocity and sarcolemma excitability remain unchanged during a sustained 10% MVIC task (Rodriguez-Falces and Place 2021). The stimulus intensity was systematically augmented by 5 mA increments until reaching a plateau in the M-wave amplitude, which represented the peak-to-peak amplitude of the sEMG. At this stage, the maximal M-wave (M_MAX_) was identified. To further validate this determination, an additional 20% increase in the current was applied, resulting in a comparable M-wave amplitude and confirming the attainment of M_MAX_. This procedure has been shown to have excellent reliability (ICC = 0.92) (Walker et al 2013). The M_MAX_ measurement was conducted at baseline and then repeated at 5 min and 30 min after the single session of RT (after rest for the control group) to examine the presence of any alteration in muscle excitability following the training session.

### Transcranial magnetic stimulation

Cortical, corticospinal, and cortico-reticulospinal excitability were assessed at baseline and then re-evaluated at 5- and 30-min post-exercise (or post-rest for the control group) using TMS, a non-invasive brain stimulation technique.

Motor evoked potentials (MEPs) were obtained from the biceps brachii of the dominant arm through TMS of the corresponding motor cortex using a circular coil connected to two Magstim 200^2^ stimulators (Magstim Co. Ltd., Whitland, Dyfed, UK). Both stimulation protocols (single-pulse and paired-pulse) were administered using identical stimulation devices to ensure standardized intensity parameters. The selected stimulation mode maintained consistent intensity levels across both single-pulse and paired-pulse protocols, thereby controlling for potential device-related variability in stimulation delivery. This methodological approach enhanced the reliability of between-protocol comparisons by eliminating intensity-related confounds that could arise from the use of different stimulation parameters or equipment (Do et al 2020).

To identify the vertex, the distances from the nasion to the inion and from the left to the right tragus were measured, and midpoint markings were placed directly on the scalp for both measurements. The intersection of these midpoints was designated as the vertex (Power & Copithorne 2013). TMS was then delivered using a Magstim 200 stimulator (Magstim Co. Ltd., Whitland, Dyfed, UK) with a 9-cm circular coil, positioned over the optimal scalp site, ~ 1.5–2 cm lateral to the vertex on the left hemisphere. The coil was held at a 45° angle to the sagittal plane, with the handle directed posteriorly, inducing a posterior-to-anterior cortical current. This orientation generated a clockwise current (viewed from above) when stimulating the left motor cortex, optimizing corticospinal activation and eliciting MEPs in the contralateral biceps brachii muscle. The ideal location, which was the point that elicited the highest peak-to-peak MEP amplitude for a given TMS intensity for eliciting biceps brachii MEPs, was identified by systematically moving the coil in small increments (0.5 cm) until the optimal response was achieved, and marked on the scalp to ensure precise coil placement throughout the experiment. In all trials, the coil was firmly held in place by the investigator, ensuring precise alignment with the scalp marking to maintain consistent stimulation accuracy. Once the motor hotspot was identified, the active motor threshold (AMT) was determined as the minimum stimulation intensity required to produce a MEP with an amplitude exceeding 200 µV in at least 5 out of 10 trials (Akalu et al 2024; Cohen et al 1998). The AMT obtained during baseline measurement was re-evaluated at 5- and 30-min post-exercise for any necessary adjustments. Five to ten trials were conducted for each test.

Corticospinal excitability was assessed using single-pulse TMS by constructing recruitment curves based on MEP amplitudes at various stimulation intensities, specifically 130%, 150%, and 170% of AMT. This method has demonstrated excellent reliability, with an ICC of 0.96 (Carson et al 2013). The cortical cSP, defined as the time from the start of the MEP to the return of uninterrupted EMG activity (Damron et al 2008; Latella et al 2017), was also quantified from the same MEP responses at each level of intensity to evaluate corticospinal inhibition. Short-interval intracortical inhibition (SICI) (GABA-_A_ mediated activity) and intracortical excitability, including intracortical facilitation (ICF) (glutamate-mediated activity) were assessed using paired-pulse TMS. SICI was triggered by administering a conditioning stimulus (CS) at 80% of the AMT and a subsequent test stimulus (TS) at 130% AMT, with a 3 ms interval between them. ICF was induced using the same stimuli but with a 10 ms interval. To reduce fatigue, a 1-min rest period was included between these tests. All TMS triggers were administered at 10-s intervals, synchronized with a metronome (Kidgell et al 2010), while participants engaged in low-level voluntary contractions at 10% of their predetermined MVIC (Rogasch et al 2009; Rossini et al 2015; Weier et al 2012). To ensure that any pre-existing variations in rmsEMG were eliminated, rmsEMG data were collected during the 100 ms interval just before delivering each TMS. Data collection and analysis were conducted using LabChart™ v 8.1.24 software (ADInstruments, Bella Vista, Australia).

### iMEP measurement

Reticulospinal cells have been shown to be effectively stimulated by TMS applied to the primary motor cortex (M1) via the cortico-reticular pathway (Fisher et al 2013). Therefore, we examined iMEP elicited by a monophasic current waveform from a figure-of-eight coil (70 mm winding diameter, D70 Alpha Flat Coated coil) connected to two Magstim 200^2^ stimulators (Magstim Co. Ltd., Whitland, Dyfed, UK). The coil was positioned tangentially to the scalp at a 45° angle to the midsagittal plane, thereby inducing a posterior-anterior current flow. Stimulation began 3 cm to the left of the vertex, moving in ~ 0.5 cm increments until the largest cMEP response was achieved at a stimulator output of 50%. Small adjustments in the anteroposterior direction (around 1–2 cm) were made to verify the location with the highest amplitude, which was then designated as the hotspot, and marked relative to the coil to ensure consistent stimulation at the same site. The cMEP hotspot was then stimulated at 100% maximum stimulatory output to induce iMEPs (Seusing et al 2024).

The participants were seated on a Nirvana Preacher Curl bench (RitFit, Memphis, TN, USA) and instructed to flex (lift the barbell towards their shoulder) and extend (lower the barbell back to the starting position) their elbows, maintaining a slow and controlled tempo of five seconds per repetition. Transcranial magnetic stimulation was then applied to elicit iMEPs in the ipsilateral biceps and cMEPs in the contralateral biceps when the target line, 110-degree elbow flexion, was reached during the concentric phase (Hu et al 2024). The real-time display of the elbow joint angle during flexion was shown on a computer screen positioned one meter in front of the participant. This was achieved using electro-mechanical goniometers (MLTS700, ADInstruments, Bella Vista, Australia) attached to the lateral side of the elbow joint, following the manufacturer’s guidelines (Biometrics 1997). Each participant completed four sets of five repetitions, with a two-minute rest interval between sets, resulting in a total of 20 stimulations. The test load was set at 30% of the participant’s unilateral 1RM. None of the participants reported experiencing fatigue or difficulty in successfully completing the total 20 repetitions.

Bilateral dynamic contractions were employed to optimize iMEP detection, as this approach has been shown to enhance RST engagement (Hu et al 2024). Altermatt et al. (2023) further demonstrated that iMEPs are more pronounced during bimanual cooperative tasks than non-cooperative tasks, supporting the use of simultaneous activation of both upper limbs to facilitate iMEP elicitation (Altermatt et al 2023).

### RFD measurement

Reticulospinal excitability was indirectly evaluated by analyzing the influence of a startling stimulus on the RFD during forceful isometric elbow flexion (Colomer-Poveda et al. 2023a; Tapia et al. 2022). Specifically, we quantified the effect of the startling stimulus on RFD during pre-planned action in response to an imperative visual signal, which is paired with either a loud, quiet, or no sound in a random order. We adhered to a previously described protocol (Colomer-Poveda et al 2023a) (Baker and Perez 2017; Sangari and Perez 2020). Participants were instructed to focus on a light-emitting diode (LED) placed one meter in front of them at eye level. Upon the LED’s illumination (20 ms), they performed an isometric elbow flexion as hard and as fast as possible. The light signal was paired with one of three auditory stimuli: a loud (startling) sound (115–120 dB, 500 Hz, 50 ms), a soft (non-startling) sound (80 dB, 500 Hz, 50 ms burst), or no sound. The sound was emitted from a speaker positioned 1 m behind the participant. The three types of sound conditions were administered in a randomized order, with a total of 60 trials conducted at an interstimulus interval of 0.2 Hz, 20 trials for each condition. Participants were familiarized with the startle stimulus (Colomer-Poveda et al 2023a; Sangari and Perez 2020) and instructed to avoid any preparatory tension or counter movements prior to the “go” signal (light illumination). Before commencing the actual testing, participants were also introduced to strong isometric biceps flexion contractions (3 repetitions) in response to the imperative visual cue. The RFD during the initial 50 ms and the subsequent 50–100 ms intervals following the onset of force, as outlined in previous studies (Akalu et al. 2024; Colomer-Poveda et al. 2023a), was then measured.

### Data analysis

The TMS and RFD data were recorded and analyzed using LabChart 8 software (ADInstruments, Bella Vista, Australia). Pre-stimulus rmsEMG activity in the dominant biceps brachii was assessed 100 ms before each TMS stimulus. Trials were repeated if the pre-stimulus rmsEMG exceeded 5 ± 1% of the maximal rmsEMG. The peak-to-peak amplitude of M_MAX_ and MEPs in the biceps brachii muscle contralateral to the cortex being stimulated was measured offline within 10–50 ms after stimulation at each intensity (130%, 150%, and 170% of AMT). To account for any peripheral changes, the biceps brachii MEPs were then normalized to M_MAX_ and scaled by a factor of 100. Additionally, maximum voluntary force (MVF) and 1-RM rmsEMG was measured during 1-RM strength assessments, and training load-volume for each participant was calculated using the formula: Training Load-Volume = sets × repetitions × load.

The duration of cSP was meticulously quantified for MEP responses at each intensity through systematic visual analysis. To ensure precision and reproducibility, the onset of the cSP was delineated from the MEP onset, while its termination was identified based on the re-establishment of sEMG activity to pre-stimulus baseline levels. This process involved defining threshold markers by placing horizontal cursors at the maximum and minimum pre-stimulus sEMG amplitudes, thereby enabling the objective determination of the exact point at which sEMG activity surpassed these reference thresholds following the cessation of cSP (Wilson et al 1993).

The total area under the recruitment curve (AURC) was determined using the trapezoidal integration method, based on data collected during the construction of recruitment curves for corticospinal excitability (MEP amplitude) in the dominant (right) biceps brachii. The experimenter remained blinded to group allocation throughout the analysis. The formula for trapezoidal integration was:

$$AURC={\sum }_{i=1}^{n-1}\frac{( {y}_{i} + {y}_{i+1})}{2}\Delta x$$

where y_i_ and y_i+1_ are the MEP amplitudes at successive points, and ($\Delta$x) is the interval between these points. This formula calculates the AURC by averaging the MEP amplitudes at two successive points, y_i_ and y_i+1_, and then multiplying by the interval width Δx. This method approximates the AURC by dividing it into a series of trapezoids. Additionally, SICI and ICF MEP amplitudes were normalized by dividing them by the MEP amplitudes of single-pulse stimulation at 130% of AMT and then multiplying by 100.

In order to determine cortico-reticular excitability, we first scrutinized the iMEP and cMEP recorded from the left and right biceps brachii muscles, respectively, during bilateral forceful biceps contraction, focusing on their latency or onset differences. A latency difference of less than 5 ms suggested the likelihood of direct stimulation of the contralateral hemisphere, rather than a true iMEP response (Ziemann et al 1999). A latency difference ranging from 5 to 13 ms has been identified as indicative of subcortical involvement (Maitland & Baker 2021; Ziemann et al 1999). Consequently, we excluded data with latency differences under 5 ms (Maitland & Baker 2021). Adopting the methodology from a prior study (Bawa et al 2004), we then analyzed the iMEP to cMEP amplitude ratio (ICAR), which provides an index of the relative excitability of the iMEP (RST) and cMEP (CST) pathways during voluntary contraction.

A customized macro in LabChart (ADInstruments, Bella Vista, Australia) was used to analyze the RFD data. Each trial was manually reviewed for potential errors in the detection of force onset by the macro, which could arise from pre-stimulus activity. Manual adjustments were made to correct any instances of incorrect force onset detection by the software. Then, the change in RFD following startling stimuli during the initial 50 ms and the subsequent 50–100 ms after the onset of force was analyzed. The onset of force (0 ms) was determined as the point when the force signal from the biceps brachii muscle exceeded 3 standard deviations from the value recorded 200 ms prior to the stimulus (Akalu et al 2024; Anzak et al 2011; Colomer-Poveda et al 2023a; Smith et al 2019).

### Statistical analysis

All statistical analyses were conducted in R (version 4.4.1; R Foundation for Statistical Computing, Vienna, Austria, 2024). The exact R environment details, including version and package specifications, can be provided for reproducibility upon request. Descriptive statistics for age, MVF, 1RM, body mass, and M_MAX_ are presented as mean ± SD.

Prior to analyses, data were screened for outliers, and the Shapiro–Wilk test was used to assess normality of data distribution. Variables that remained non-normal despite transformations (VA, ICF, RFD, cSP at 130% AMT, and rmsEMG) were analyzed using a Generalized Linear Mixed Model (GLMM). For data that were normally distributed (cSP at 170%) or successfully transformed using log transformation (AURC, cSP at 150, ICAR) and Box-Cox transformation (AMT, and SICI), a transformation technique that can handle positive skewness by adjusting according to the data’s skewness, a linear mixed model for repeated measures (LMM_RM_) was employed. These models examined changes in cortical (SICI and ICF), corticospinal (SP and CSE), cortico-reticulospinal (ICAR), and reticulospinal (RFD) excitability across groups and time. Factors included type of RT (MP-RT, SP-RT, and control) and time (Pre, Post 5, and Post 30 min), with additional factors for RFD data: sound type (loud, soft, no sound) and RFD interval (first 50 ms, subsequent 50–100 ms). Both models accounted for fixed and random effects, managing potential missing data points and baseline variability (Wilkinson et al 2023). Time (pre, post 5, post 30) and types of RT (MP-RT, SP-RT, and Control) were treated as fixed effects, while subjects were treated as a random variable. Sound conditions (loud, soft, and no sound), time, and time intervals (first 50 ms and 50–100 ms) were treated as repeated measured factors.

To determine baseline differences among groups, a one-way analysis of variance (ANOVA) was conducted on the dependent variables: MVF, 1RM, VA, AURC, ICF, SICI, SP, RFD, and ICAR. Post-hoc analyses were performed using the Bonferroni correction approach. Fixed effect estimates were used to examine the magnitude of the effect of each independent variable on the dependent variable, accounting for the influence of other variables. For log-transformed data, estimates (β) were back-transformed to their original scale using the exponential function (*e*^β^) to facilitate meaningful interpretation. Generalized Eta Squared (η^2^G), which accounts for both fixed and random effects (Olejnik & Algina 2003), was used to measure the effect sizes of group, time, type of sound, and their interaction on the outcome variables. Effect sizes were categorized as small (η^2^G ≥ 0.01), medium (η^2^G ≥ 0.06), and large (η^2^G ≥ 0.14) (Cohen 2013). The test–retest reliability of our measurements across three time points was evaluated using Intraclass Correlation Coefficient (ICC) values for all outcome variables within the control group. Statistical significance was set at α = 0.05 and only significant comparisons are reported.

## Results

### Baseline characteristics, Maximal voluntary force and test–retest Reproducibility

A total of 36 participants (12 per group), matched for age and sex with no prior RT history, were included in this study. The mean age was 27 ± 6, 28 ± 6, and 29 ± 4 years for the control, MP-RT, and SP-RT groups, respectively (F(1,34) = 0.67, *p* = 0.42). Baseline comparisons revealed no significant differences among groups for age (p = 0.420), body mass (*p* = 0.78), maximum voluntary force (*p* = 0.64), 1RM (*p* = 0.71), M_MAX_ (*p* = 0.93), AMT stimulus intensity (*p* = 0.48), AMT MEP amplitude (*p* = 0.16), ICAR (*p* = 0.831), AURC (*p* = 0.980), SICI (*p* = 0.65), ICF (*p* = 0.48), and cSP (*p* = 0.710). Pre-stimulus rmsEMG values for single-pulse and paired-pulse TMS remained consistent across baseline and post-exercise measurements within groups (*p* > 0.05) **(**Table 1**)**.

**Table 1:** Mean ± SD for demographic, AMT, M_MAX_, and TMS-derived rmsEMG variables before and after MP and SP resistance training in the biceps brachii

Variables	Control (*n* = 12)			Metronome-paced (*n* = 12)			Self-paced (*n* = 12)		
	Baseline	Post-05	Post-30	Baseline	Post-05	Post-30	Baseline	Post-05	Post-30
Age (years) #	27 ± 6.82	N/A	N/A	28.17 ± 6.56	N/A	N/A	29 ± 4.55	N/A	N/A
Body mass (kg)#	68.25 ± 14	N/A	N/A	72.12 ± 13	N/A	N/A	70.08 ± 12	N/A	N/A
1RM (kg)#	13.33 ± 3.56	N/A	N/A	13.16 ± 3.99	N/A	N/A	14.32 ± 3.54	N/A	N/A
1RM rmsEMG (%M_MAX_) #	5.22 ± 1.18	N/A	N/A	5.76 ± 1.47	N/A	N/A	5.78 ± 2.31	N/A	N/A
Training Load-Volume (Au)*#	N/A	N/A	N/A	237 ± 71.7	N/A	N/A	344 ± 85.0	N/A	N/A
MVF (N)	98.75 ± 36.35	87.33 ± 32	79.13 ± 30	92.28 ± 42	78.61 ± 24	71.83 ± 19	106.50 ± 31	83.88 ± 31	87.06 ± 31
MVF rmsEMG (%M_MAX_)	5.13 ± 3.65	4.84 ± 1.86	4.79 ± 2.14	6.84 ± 2.32	6.64 ± 1.94	6.56 ± 2.21	5.25 ± 2.75	4.44 ± 2.10	5.02 ± 2.10
SP rmsEMG (%M_MAX_)	0.90 ± 0.39	0.84 ± 0.39	0.89 ± 0.38	0.80 ± 0.41	0.98 ± 0.47	1.13 ± 0.54	0.93 ± 0.57	0.94 ± 0.53	1.09 ± 0.62
PP rmsEMG (%M_MAX_)	0.96 ± 0.47	0.89 ± 0.49	0.97 ± 0.60	0.92 ± 0.53	0.92 ± 0.41	0.96 ± 0.41	0.90 ± 0.40	0.88 ± 0.54	0.96 ± 0.56
M_MAX (_mV)	12.48 ± 4	12.45 ± 4	12.54 ± 4	11.88 ± 4	11.58 ± 4	11.39 ± 4.50	12.06 ± 3	12 ± 2.86	11.86 ± 3
AMT SI (%)	40 ± 10	39.58 ± 10	39.67 ± 10	40.83 ± 8.20	39.83 ± 7.80	39.17 ± 7.50*	37 ± 4.79	36.25 ± 4.10	36.25 ± 5.30

*AMT* active motor threshold, *Au* Arbitrary unit, *kg* kilogram, *mA* milliampere, *M*_*MAX*_ maximum compound action potential, *mV* milli volt, *MVF* maximum voluntary force, *PP* Paired-pulse, *rmsEMG* root-mean square electromyography, *RM* repetition maximum, *SI* stimulator intensity, *SP* single-pulse

^#^Single time measurements; *Statistically significant at *p* < 0.05;

Post-exercise, a significant main effect of Time for MVF was observed at Post 30 (*β* = − 0.23, SE = 0.07, *t* = − 3.07, *p* = 0.002), but not at Post 5 min (*β* = − 0.13, SE = 0.07, *t* = − 1.68, *p* = 0.093). No significant main effects were found for Group (MP-RT: *β* = − 0.09, SE = 0.20, *t* = − 0.50, *p* = 0.618; SP-RT: *β* = 0.09, SE = 0.20, *t* = 0.46, *p* = 0.648), nor were there significant Time × Group interactions (all *p* > 0.05) **(**Table 1**)**.

Test–retest reproducibility within the control group demonstrated excellent reliability for multiple measures: cSP at 170% AMT (ICC = 0.98, 95% CI [0.96, 0.99]), ICAR (ICC = 0.97, 95% CI [0.94, 0.98]), and AURC (ICC = 0.91, 95% CI [0.87, 0.94]). Good reliability was observed for SICI (ICC = 0.83, 95% CI [0.77, 0.87]). Moderate reproducibility was found for ICF (ICC = 0.50, 95% CI [0.42, 0.57]). Rate of force development maintained high consistency under both visual stimuli (ICC = 0.94, 95% CI [0.91, 0.96]) and visual startling stimuli (ICC = 0.93, 95% CI [0.90, 0.95]) conditions (Table 2).

**Table 2:** Test–retest reliability (ICC) of outcome variables in the control group

Outcome variable		ICC value	95% CI and *p*-value
AURC (Au)		0.91	95% CI [0.78, 0.97], *p* < 0.001
ICAR (Au)		0.97	95% CI [0.94, 0.99], *p* < 0.001
ICF (%test response)		0.50	95% CI [0.13, 0.78], *p* = 0.003
SICI (%test response)		0.83	95% CI [0.62, 0.94], *p* < 0.001
RFD (Ns-1)	VS	0.94	95% CI [0.85, 0.98], *p* < 0.001
	VnSS	1.00	95% CI [1.00, 1.00], *p* < 0.001
	VSS	0.93	95% CI [0.84, 0.98], *p* < 0.001
SP (ms)	130%-AMT	0.97	95% CI [0.92, 0.99], *p* < 0.001
	150%-AMT	0.88	95% CI [0.73, 0.96], *p* < 0.001
	170%-AMT	0.98	95% CI [0.94, 0.99], *p* < 0.001

*AURC* area under recruitment curve, *Au* arbitrary unit, *CSP* cortical silent period, *ICAR* ipsilateral to contra lateral motor evoked potential amplitude ratio, *ICC* intraclass correlation coefficient, *ICF* intra cortical facilitation, *Ns-1* Newton per second, *ms* milliseconds, *SICI* short-interval cortical inhibition, *RFD* rate of force development, visual stimuli, *VnSS* visual non-startling stimuli, *VSS* visual startling stimuli

### Corticospinal excitability and inhibition

#### Cortical excitability

The analysis of AMT stimulator intensity revealed no significant main effects for Time (Post 5: *β* = − 0.01, SE = 0.012, *t* = − 0.83, *p* = 0.410; Post 30: *β* = -0.02, SE = 0.012, *t* = -1.67, *p* = 0.099) or Group (MP-RT: *β* = 0.02, SE = 0.034, *t* = 0.59, p = 0.559; SP-RT: *β* = 0.01, SE = 0.034, *t* = 0.29, *p* = 0.771). However, a significant interaction effect emerged for the MP-RT group at Post 30 min (*β* = − 0.03, SE = 0.012, *t* = − 2.64, *p* = 0.008, η^2^G = 0.22). The interaction between Time and Group explained 22% (large effect) of the total variance in AMT (η^2^G = 0.22). Post hoc comparisons revealed a significant decrease in AMT (∼4.08 ± 7.9%) from Baseline to Post 30 for the MP-RT group only (EMD = 0.04, SE = 0.01, *z* = 4.70, *p* < 0.001).

**Fig. 2:**
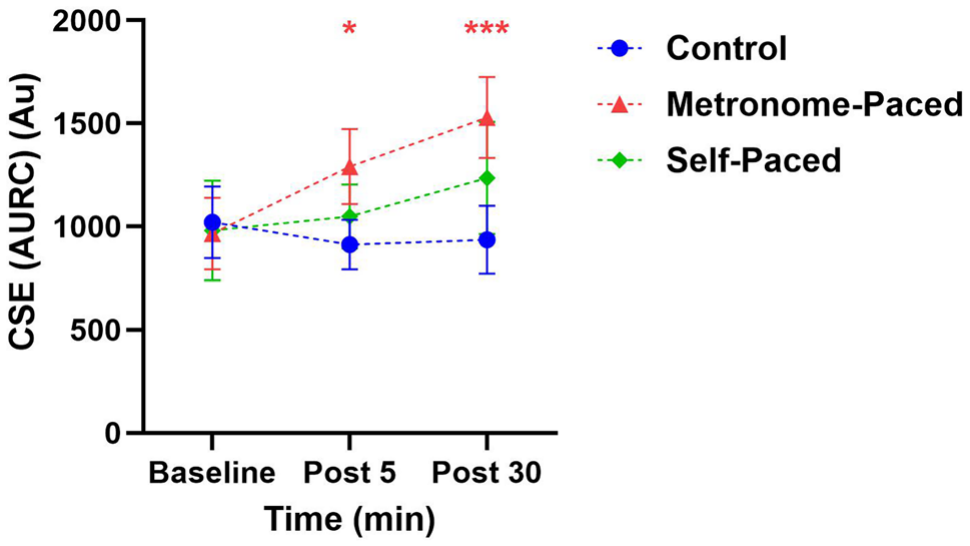
CSE(AURC) at baseline, post 05 and post 30 min following a single bout of MP and SP resistance exercise. *Statistically significant at *p* < 0.05, **Statistically significant at *p* < 0.001, all p-values are as compared to the baseline. *Au* Arbitrary unit; *AURC* area under recruitment curve; *CSE* corticospinal excitability; *MP* metronome-paced, *SP* self-paced

The AURC analysis showed significant Group × Time interactions, with the MP-RT group demonstrating increased AURC at both Post 5 (β = 0.41, SE = 0.16, t(63.87) = 2.45, *p* = 0.016) and Post 30 (*β* = 0.54, SE = 0.17, t(63.53) = 3.23, *p* < 0.001, η^2^G = 0.69). These changes represented approximately 50% and 72% increases in AURC at Post 5 and Post 30, respectively. The interaction between Time and Group explained 69% (large effect) of the total variance in AURC (η^2^G = 0.69). Post hoc analysis confirmed significant differences between MP-RT and control groups at Post 30 (EMD = 0.50, SE = 0.13, t (63.4) = 4.05, *p* = 0.004) (Fig. 2**, **Table 3**).**

**Table 3:** Mean ± SD values for cSP, and TMS /MEP amplitudes measured before and after a single bout of MP and SP resistance exercise in the biceps brachii

Variable		Control (*n* = 12)			Metronome-paced (*n* = 12)			Self-paced (*n* = 12)		
		Baseline	Post-05	Post-30	Baseline	Post-05	Post-30	Baseline	Post-05	Post-30
AURC (CSE) (Au)		1021.45 ± 602	912.19 ± 417	935.13 ± 573	965.19 ± 579	1290.55 ± 630*	1431 ± 638***	981.31 ± 836.32	1049.71 ± 540	1235.94 ± 942
cSP (ms)	130% AMT	74.88 ± 28	74.05 ± 27	71.23 ± 27	85.95 ± 4.67	78.36 ± 13	66.54 ± 16	83.79 ± 22	69.5 ± 19.8	63.36 ± 25
	150% AMT	99.64 ± 30	104.84 ± 32	101.53 ± 32	99.52 ± 11	92.92 ± 17*	82.52 ± 17***	98.72 ± 17	86.15 ± 18**	87.92 ± 19*
	170% AMT	126.6 ± 35	123.72 ± 37	124.6 ± 36	121.64 ± 19	102.68 ± 24*	92.37 ± 20***	114.54 ± 28	98.79 ± 19	90.67 ± 22.27**
SICI (%test response)		73.05 ± 14	74.16 ± 17	73.54 ± 17	68.94 ± 13	80.99 ± 17	93.35 ± 10***	73.23 ± 11	77.74 ± 14	71 ± 21
ICF (%test response)		115 ± 14.90	120 ± 16	119 ± 13	115 ± 29	135 ± 27	145 ± 39*	127 ± 37	121 ± 20	117 ± 9
ICAR		0.27 ± 0.15	0.27 ± 0.16	0.27 ± 0.16	0.32 ± 0.19	0.28 ± 0.11	0.29 ± 0.12	0.27 ± 0.19	0.45 ± 0.26***	0.52 ± 0.25***

*AU* arbitrary unit, *AURC* area under recruitment curve, *cSP* cortical silent period, *CSE* corticospinal excitability, *ICAR* Ipsilateral to contra lateral motor evoked potential amplitude ratio, *ICF* intra-cortical facilitation, milliseconds; *mV* millivolt, *SICI* Short-interval cortical inhibition

*Statistically significant at *p* < 0.05; **Statistically significant at *p* < 0.01; *** Statistically significant at *p* < 0.001

#### Cortical Silent Period

**Fig. 3:**
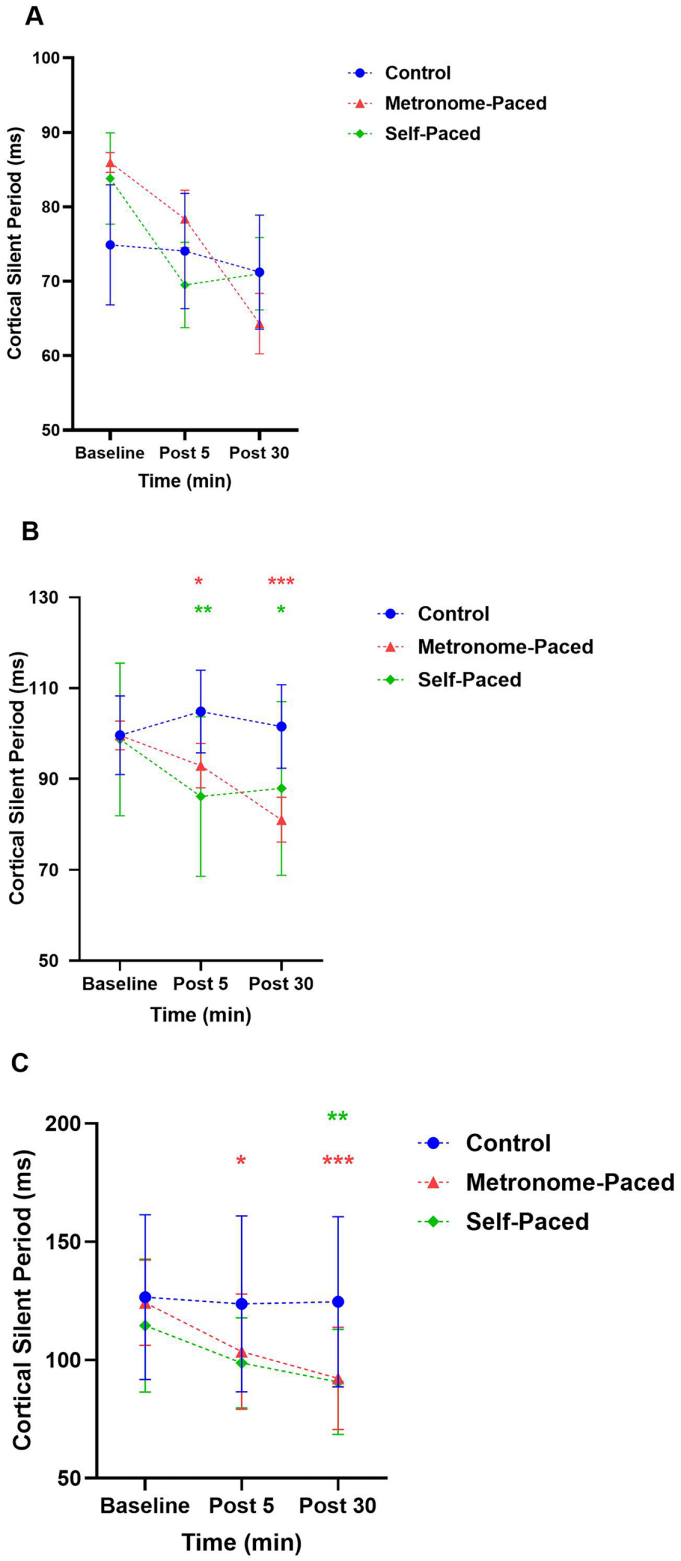
cSP at 130% AMT (**A**), 150% AMT (**B**), and 170% AMT (**C**) at post 05 and post 30 min following a single bout of MP and SP resistance exercise. *AMT* active motor threshold, *MP* metronome-paced, *SP* self-paced, *SP* silent period. **Statically significant at *p* < 0.01, ***Statistically significant at *p* < 0.001. *p*-values are as compared to the baseline

Analysis at 130% AMT revealed no significant main effects or interactions across time points or groups (MP-RT × Post 5: *β* =  − 0.09, SE = 0.13, t(66) =  − 0.72, *p* = 0.47; SP-RT × Post 5: *β* =  − 0.18, SE = 0.13, t(66) =  − 1.37, *p* = 0.17; MP-RT × Post 30: *β* =  − 0.23, SE = 0.13, t(66) =  − 1.74, *p* = 0.08; SP-RT × Post 30: *β* =  − 0.24, SE = 0.13, t(66) =  − 1.85, *p* = 0.06) (Fig. 3a). At 150% AMT, both intervention groups showed significant changes. The MP-RT group demonstrated decreased cSP at Post 5 (*β* = − 0.13, SE = 0.06, t(66) =  − 2.10, *p* = 0.040) and Post 30 (*β* = − 0.21, SE = 0.06, t(66) = − 3.51, *p* < 0.001, η^2^G = 0.75), corresponding to ∼12% and ∼20% reductions, respectively (Fig. 3b). Similarly, the SP-RT group showed reductions of ∼17% at Post 5 (β = − 16.1, SE = 7.10, t(66) =  − 2.30, *p* = 0.03) and ∼13% at Post 30 (*β* = − 0.14, SE = 0.06, t(66) =  − 2.28, *p* = 0.026). At the 170% AMT, a significant Group × Time interaction emerged for the MP-RT group at Post 5 (β = − 16.1, SE = 7.10, t(66) =  − 2.30, *p* = 0.03), and at Post 30 (*β* = − 27.25, SE = 7.15, t(66) =  − 3.81, *p* < 0.001, η^2^G = 0.98), demonstrating a ∼15.6 ± 13.5% and 23.8% ± 12.2% reduction in cSP at Post 5 and Post 30, respectively, compared to the control group **(**Fig. 3**c).** Similarly, there was a decrease in cSP at Post 30 (*β* = − 21.87, SE = 7.15, t(66) = − 3.10, *p* < 0.003) in the SP-RT group, corresponding to an ∼18.9 ± 19% decrease in cSP. Post hoc analysis confirmed a significant increase from baseline to Post 30 in both the MP-RT (EMD = 29.2, t(66) = 5.7, *p* < 0.001) and SP-RT (EMD = 23.8, t(66) = 4.723, p < 0.001) groups (Supplementary Fig. 1).

#### Intracortical excitability

**Fig. 4:**
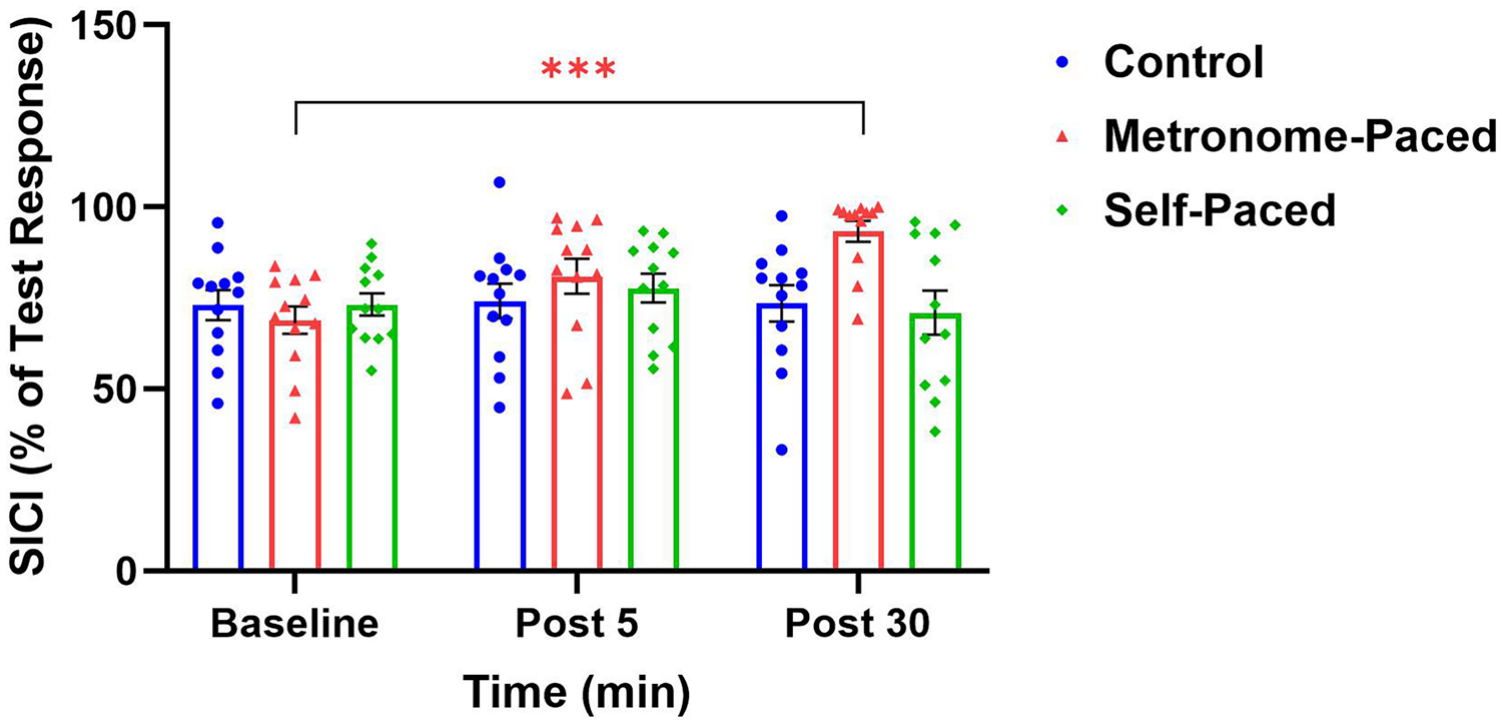
SICI following MP and SP resistance exercise at Post 05 and post 30. ***Statistically significant at *p* < 0.001, as compared to the baseline. *MP* metronome-paced; *SP* self-paced; *SICI* short interval cortical inhibition

SICI analysis revealed a significant Group × Time interaction (*β* = 1.56, SE = 0.38, t (66.0) = 4.14, *p* < 0.001, η^2^G = 0.89), with the MP-RT group showing a ∼35.4 ± 7.6% reduction in SICI at Post 30. A large proportion of the variance in SICI (89%) was attributable to the interaction between Time and Group (η^2^G = 0.89). Post hoc analyses confirmed significant decreases in SICI for the MP-RT group compared to both control (EMD = 1.37, SE = 0.38, t (66.2) = 3.63, *p* = 0.015) and SP-RT groups (EMD = 1.38, SE = 0.38, t(66.2) = 3.67, *p* = 0.014) at Post 30. Within-group comparisons for the MP-RT group showed a significant decrease in inhibition from Baseline to Post 30 (EMD = 1.63, SE = 0.27, t (66.0) = 6.08, *p* < 0.001) (Fig. 4).

ICF demonstrated a marginally significant interaction for MP-RT at Post 30 (*β* = 0.13, SE = 0.07, t = 1.94, *p* = 0.052, η^2^G = 0.99). Post hoc testing revealed a significant increase from Baseline to Post 30 in the MP-RT group (EMD = 0.17, SE = 0.05, *z* = 3.46, *p* = 0.016).

### Subcortical excitability and behavioral measures

#### Reticulospinal and cortico-reticulospinal excitability

**Fig. 5:**
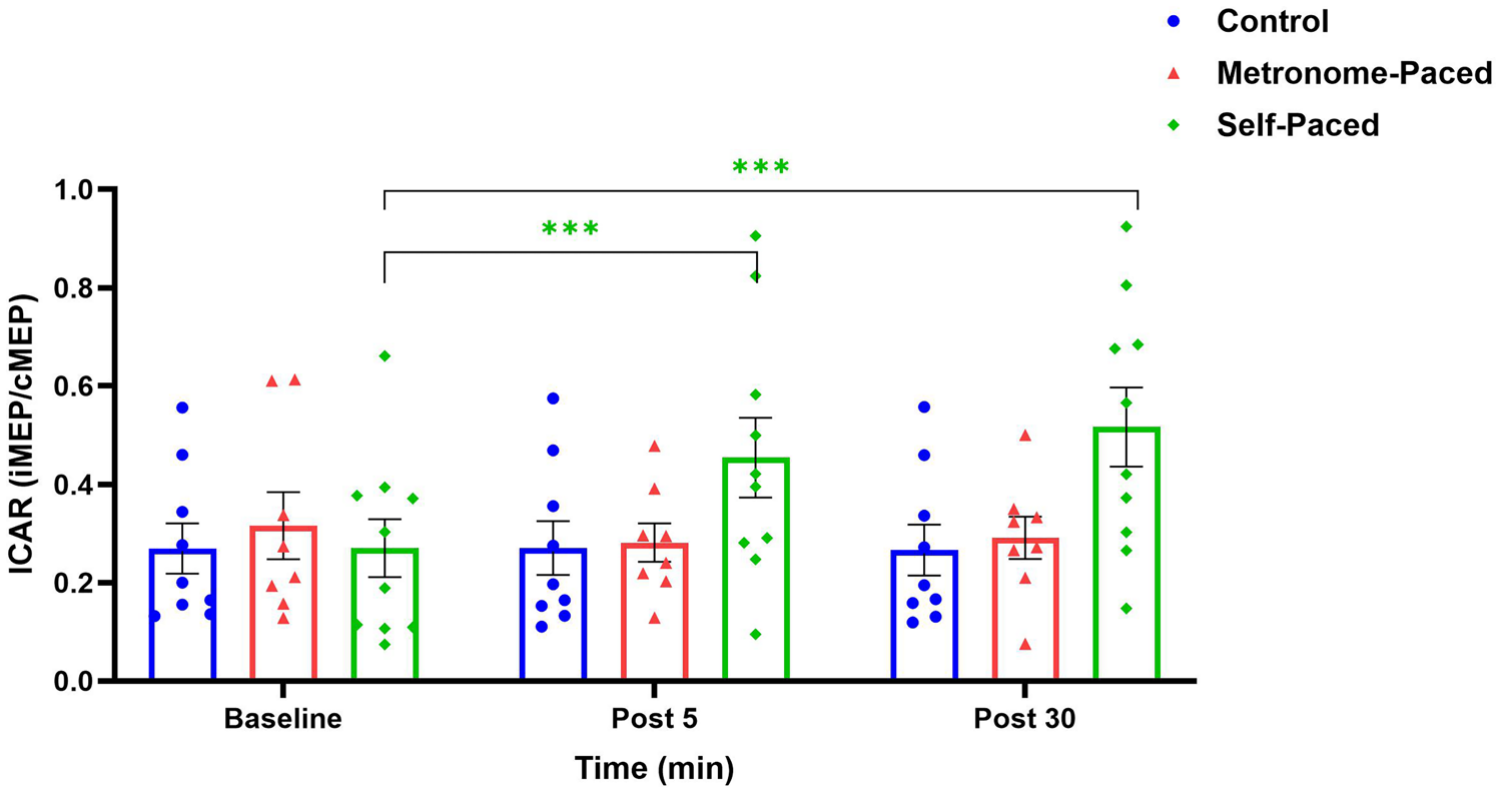
ICAR following MP and SP resistance exercise at Post 05 and post 30-min. ICAR: Ipsilateral to contralateral motor evoked potential amplitude ratio (ICAR = iMEP amplitude/cMEP amplitude); *MP* metronome-paced; *SP* self-paced. **Statistically significant at *p* < 0.001 as compared to baseline

ICAR analysis revealed significant interaction effects for SP-RT at both Post 5 (*β* = 0.59, SE = 0.13, t (48) = 4.57, *p* < 0.001, η^2^G = 0.84) and Post 30 (*β* = 0.76, SE = 0.13, t (48) = 5.8, *p* < 0.001, η^2^G = 0.84), corresponding to 80.3% and 114% increases, respectively. The interaction between Group and Time accounted for a substantial proportion of variance in ICAR, with 84% (large effect) of the total variability attributed to differences in training effects over time. Post hoc testing showed significant increases from baseline in the SP-RT group at both Post 5 (EMD = 0.58, SE = 0.09, t (48) = 6.5, *p* < 0.001) and Post 30 (EMD = 0.75, SE = 0.09, t (48) = 8.3, *p* < 0.001) (Fig. 5) (Supplementary Fig. 2).

**Fig. 6:**
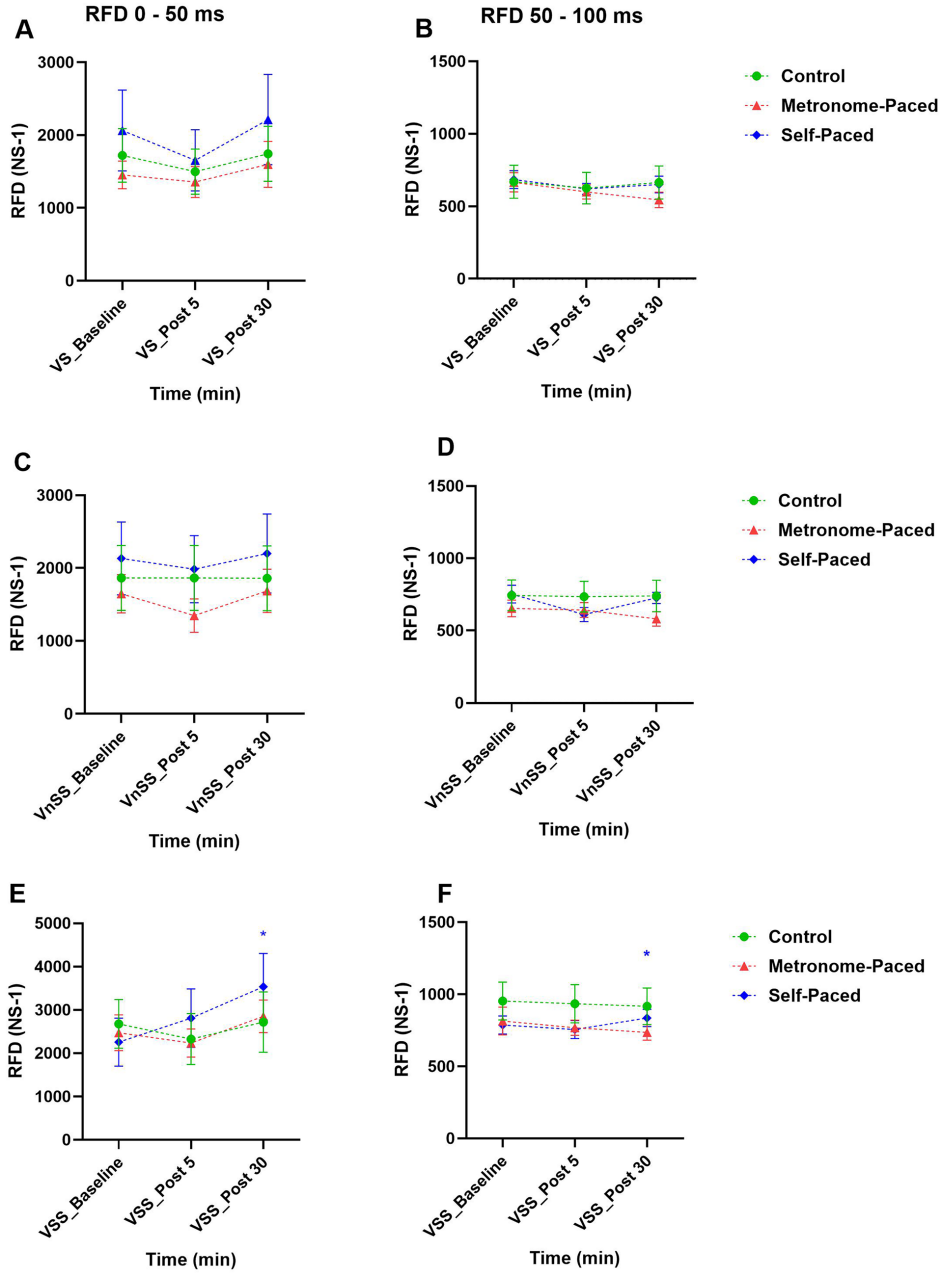
RFD during the first 50 ms (**A**, **C**, **E**) and subsequent 50–100 ms (**B**, **D**, **F**) following MP and SP resistance exercise at Baseline, Post 05, and Post 30 during visual stimuli (**A** and **B**), visual non-startling stimuli (**C** and **D**), and visual startling stimuli (**E** and **F**). *MP* metronome-paced, *SP* self-paced; *RFD* rate of force development, *NS*^*−1*^ Newton per second, *ms* millisecond; *VS* visual stimuli, *VnSS* visual non-startling stimuli, *VSS* visual startling stimuli; *Statistically significant at *p* < 0.05, as compared to the baseline

RFD analysis revealed significant main effects for both Sound condition (Startling vs. Visual stimuli: *β* = 0.45, SE = 0.11, t = 4.00, *p* < 0.001, η^2^G = 0.02) and Interval (0–50 ms vs. 50–100 ms: *β* = 0.79, SE = 0.15, *t* = 5.17, *p* < 0.001). A significant three-way interaction emerged for SP-RT × Post 30 × Startling stimuli (*β* = 0.468, SE = 0.23, *t* = 2.06, *p* = 0.039, η^2^G = 0.01), with the SP-RT group showing a 56 ± 64% increase in RFD at Post 30 under startling stimuli conditions (Fig. 6a). For the 50–100 ms interval, startling stimuli showed a significant main effect compared to visual stimuli (*β* = 0.36, SE = 0.08, *t* = 4.46, *p* < 0.001, η^2^G = 0.02). Post hoc analysis revealed a ∼6.1 ± 2.3% increase in RFD at Post 30 during startling stimuli for the SP-RT group (EMD = 0.47, SE = 0.11, *z* = 4.16, *p* = 0.001) (Fig. 6b, Table 4).

**Table 4:** Mean ± SD for RFD during the first 50 ms and the subsequent 50–100 ms following three conditions (VS, VnSS and VSS) after a single session of MP and SP resistance exercise for the biceps brachii

Variables	Interval	Type of stimuli	Control (*n* = 12)			Metronome-paced (*n* = 12)			Self-paced(n = 12)		
			Baseline	Post-05	Post-30	Baseline	Post-05	Post-30	Baseline	Post-05	Post-30
RFD (NS^−1^)	0–50 ms	VS	1721.67 ± 1278	1497.83 ± 1078	1742.5 ± 1316	1452.93 ± 661	1356.96 ± 739	1599 ± 1094	2064 ± 1921	1652.75 ± 1458	2213.25 ± 2148
		VnSS	1863.93 ± 1542	1863.43 ± 1541	1859.35 ± 1539	1646.76 ± 907	1345.76 ± 799	1687.22 ± 1036	2130 ± 1735	1982.67 ± 1593	2199.5 ± 1885
		VSS	2678.04 ± 1958	2327.64 ± 2033	2718.96 ± 2413	2472.12 ± 1434	2233.81 ± 1125	2853.28 ± 1308	2255.83 ± 1918	2812.33 ± 2340	3534.25 ± 2666 *
	50–100 ms	VS	669.56 ± 394	625.89 ± 376	664.39 ± 396	665.1 ± 231	599.68 ± 169	544.98 ± 186	684.08 ± 213	621.17 ± 130	650.25 ± 202
		VnSS	743.14 ± 369	734.56 ± 370	739.89 ± 378	653.58 ± 202	641.52 ± 177	580.19 ± 174	751.42 ± 212	610.75 ± 168	725.58 ± 136
		VSS	953.08 ± 455	934.83 ± 461	916.42 ± 438	814.06 ± 335	768.42 ± 184	735.62 ± 187	787.25 ± 219	755.25 ± 217	835.42 ± 208 *

*ms* milliseconds, *(NS*^*−1*^*)* Newton per second, *M*_*MAX*_ maximum compound action potential, *Hz* Hertz, *RFD* Rate of force development, *rmsEMG* root-measure square electromyography; visual stimuli, *VnSS* visual non-startling stimuli, *VSS* visual startling stimuli

*Statistically significant at *p* < 0.05

## Discussion

This study investigated the acute effects of a single session of RT on cortical, corticospinal, cortico-reticular, and reticulospinal pathways. Additionally, it aimed to determine whether MP-RT versus self-paced SP-RT differentially influences these neural pathways. Our findings demonstrated that MP-RT significantly increased cortical and corticospinal excitability while reducing intracortical inhibition, thereby enhancing cortical responsiveness and decreasing cortical inhibition post-training. In contrast, SP-RT led to modulation of cortico-reticulospinal and reticulospinal pathways, suggesting that SP-RT may be more effective in facilitating neural adaptations at the subcortical level. These results underscore the distinct sites of neural adaptation influenced by MP-RT and SP-RT. Specifically, MP-RT appears to favor enhancements in cortical and CST excitability, whereas SP-RT contributes to increased cortico-reticulospinal and RST excitability**.**

### Intracortical and corticospinal changes

MP-RT, unlike SP-RT, increased AURC (calculated from MEP amplitudes), reflecting enhanced CST excitability, and decreased SICI at 30 min post-training. Additionally, acute increases in CSE and reductions in SICI following MP-RT, but not SP-RT, have been documented (Leung et al 2015), consistent with our results. The increased CSE and reduced SICI after a single session of MP-RT also align with findings from another study demonstrating similar effects (Mason et al 2019a). In contrast, the absence of changes in CSE and SICI following SP-RT is in line with other research reporting no significant modulation of these measures after a single session of SP-RT (Hortobágyi et al 2011; Latella et al 2017). Overall, the cortical and corticospinal findings for SP-RT from our study are consistent with previous research involving similar SP training protocols (Chen et al 2006; Latella et al 2018, 2017). These studies reported no significant changes in intracortical measures (SICI and ICF) (Latella et al 2018) and suggested that acute neurophysiological adaptations to SP-RT occur outside the primary motor cortex (M1) and CST (Chen et al 2006; Latella et al 2017). Furthermore, a chronic RT study in non-human primates, macaque monkeys, demonstrated neural adaptations primarily in intracortical and reticulospinal circuits, while corticospinal adaptations were less prominent (Glover and Baker 2020). Specifically, muscle responses to M1 and RST stimulation increased post-training, whereas CST responses showed no significant change. Several studies have examined neural adaptations associated with chronic RT, providing a broader context for interpreting our acute findings. Notably, these studies align with our results, showing that MP-RT enhances MEP amplitude and reduces SICI, whereas SP-RT does not elicit comparable changes in MEPs or SICI (Leung et al 2017; Manca et al 2016; Weier et al 2012). Our findings are further supported by a recent systematic review (Gordon et al 2024), indicating that tempo-controlled RT (i.e., externally paced RT) consistently enhances CSE and reduces SICI compared to traditional, SP-RT. Notably, this review reported that in 100% of included studies, SP-RT elicited no change in CSE or SICI (Gordon et al 2024).

The reduction in SICI observed following MP-RT appears important for the early enhancement of synaptic efficacy. This effect is likely attributable to altered γ-aminobutyric acid (GABA) receptor efficacy, resulting in decreased GABA-mediated inhibition (Hess et al 1996; Hess and Donoghue 1994; Kujirai et al 1993). However, paired-pulse inhibition during background contraction may not be exclusively attributed to intracortical or GABAergic inhibition, as spinal mechanisms could also contribute. During muscle contraction, increased spinal motoneuron excitability enhances responsiveness to the initial (I1 wave) descending volley induced by TMS, which is typically unaffected by SICI (Hanajima et al 1998). This heightened responsiveness results in larger conditioned MEPs compared to the relaxed state, reducing the apparent magnitude of SICI (Ortu et al 2008). Notably, the GABAergic circuit primarily modulates late I3 waves, suggesting that multiple mechanisms underlie paired-pulse inhibition (Di Lazzaro et al 1998; Ilić et al 2002). Thus, SICI measured during background contraction at 80% AMT may not be purely cortical but may also reflect spinal excitability changes.

The increase in CSE following a single session of MP-RT is considered a key contributor to progressive strength gains. This is supported by a study showing attenuated strength improvements when repetitive TMS (rTMS) was applied to the primary motor cortex (M1) during RT (Hortobágyi et al 2009). rTMS accompanied both reduced excitability and diminished strength gains. Although the exact mechanisms remain unclear, these effects may involve distinct neural pathways that require further investigation.

Some studies have proposed that the increased excitability and decreased inhibition observed following RT may serve as compensatory mechanisms to counteract peripheral fatigue (Latella et al 2016, 2017). However, fatigue is unlikely to have influenced the results in the present study. A 5-min rest period was provided to minimize the potential effects of fatigue, and RFD, which is known to be highly sensitive to fatigue, showed no significant decrease, suggesting minimal fatigue. Additionally, no significant reductions in MVF and M_MAX_ were observed, further supporting the absence of fatigue. In contrast, studies supporting the compensatory mechanism hypothesis have often reported reduced M_MAX_ as an indicator of fatigue.

In the present study, the most significant changes in intracortical inhibition, corticospinal excitability, and cortico-reticulospinal pathways were observed at 30 min post-exercise, but not at 5 min. Although efforts were made to minimize fatigue and no significant reductions were observed in RFD and M_MAX_, both sensitive markers of fatigue, it is plausible that residual fatigue transiently suppressed early neurophysiological responses, delaying their manifestation until 20–30 min post-exercise (Carroll et al 2017). This interpretation aligns with findings by Weavil and Amann (2018), who proposed that fatigue may initially obscure neuromodulatory effects (Weavil and Amann 2018).

Interestingly, the emergence of these changes at 30 min occurred earlier than reported in previous studies, which have observed more delayed neurophysiological responses following RT (Latella et al 2016). A potential explanation for this accelerated response may be the circadian timing of our intervention. For all participants, training was conducted in the afternoon, a period associated with elevated core body temperature and peak neuromuscular performance, both factors known to enhance corticospinal excitability (Douglas et al 2021; Teo et al 2011). These physiological conditions may have facilitated the earlier onset of neuroplastic responses in our participants.

Regarding the duration of these responses, prior research suggests that acute increases in corticospinal excitability and reductions in intracortical inhibition can persist from approximately 25 min to several hours, and in some cases up to 72 h, depending on exercise type and intensity (Latella et al 2016, 2017; Nuzzo et al 2016; Singh et al 2014). While the current study was limited to a 30-min post-exercise observation period, further research is warranted to determine the longevity of these neurophysiological changes and their potential contribution to long-term plasticity.

The increased CSE and decreased SICI observed in the MP-RT group, along with the lack of change in the SP-RT group, may be attributed to the distinctive demands of each training type. Non-human primate studies suggest distinct roles for the corticospinal and RSTs in modulating muscle contraction force during weight-lifting tasks, a mechanism likely relevant to humans as well (Glover and Baker 2022). Neural recordings from corticospinal neurons in the M1 and reticulospinal neurons in the reticular formation at varying force levels reveal that these tracts encode force differently. Reticulospinal cells provide a consistent, generalized signal, while corticospinal neurons exhibit a more nuanced activity pattern, supporting fine motor adjustments (Glover and Baker 2022). MP-RT requires precise timing, coordination, and synchronization with the metronome, engaging more extensive neural pathways, while SP-RT primarily involves force application with less emphasis on timing and likely had a little impact on cortical and CST (Ackerley et al 2007; 2011). Manipulating the tempo of movements, as in MP-RT, imposes precise control over the speed of each repetition, providing targeted challenges to the M1. This controlled pacing enhances motor control and performance, engaging motor regions such as the sensorimotor cortex, premotor cortex (PMC), and supplementary motor areas (SMA and pre-SMA) (Gordon et al 2024). Such precise, slower repetitions likely demand greater accuracy to maintain the specific timing, strengthening existing neural connections and promoting use-dependent plasticity through reduced local inhibition (Ackerley et al 2011; Gordon et al 2024). Moreover, synchronizing movement with auditory cues, such as a metronome, further reinforces selective cortical activation, enhancing corticospinal excitability (Chen et al 2006; Jäncke et al 2000). The synchronized arrival of auditory input from the metronome, relayed through the auditory cortex to the premotor and supplementary motor areas, aligns with the depolarization of pyramidal cells in the M1 during movement execution (Jantzen et al 2009). This convergence, grounded in Hebbian principles, may increase synaptic efficacy and support more consistent and selective motor neuron recruitment (Morris 1999). MP-RT, therefore, likely creates a spatially selective bias in corticospinal excitability, a pattern less evident in SP-RT (Morris 1999). Notably, task-specific activation patterns have been observed in MP skill training but not in SP skill training (Ackerley et al 2011; Perez et al 2004). MP movements selectively activate targeted cortical areas, whereas SP training engages a broader network of cortical and subcortical regions, as identified by functional brain imaging (Ackerley et al 2011). Another plausible mechanism underlying the observed alterations in corticospinal excitability and inhibition involves sensory feedback, particularly from group III muscle afferents. The externally paced nature of MP-RT may enhance sensorimotor feedback by enforcing a consistent movement rhythm, in contrast to SP-RT, where participants regulate their own pacing. This structured timing could modulate afferent input from proprioceptive and mechanoreceptive sources, potentially contributing to the observed neurophysiological responses (Hortobágyi et al 1997; Howatson et al 2011; Kidgell et al 2015). Understanding these neural responses could inform the design of rehabilitation programs that leverage these rapid adaptations, enabling clinicians to optimize recovery strategies for patients with musculoskeletal or neurological challenges.

In this study, decreased cSP durations at 150% and 170% of AMT stimulation were observed following both types of RT, suggesting a release or reduction of inhibition. This aligns with previous studies that reported decreased cSP duration after a single session of RT (Latella et al 2017) as well as findings from a short-term lower-limb training study (Christie and Kamen 2014). Additionally, a separate study reported a reduction in cSP duration following RT in male participants (Latella et al 2018). However, cSP at 130% AMT in our study showed no significant changes, indicating that cSP duration at lower stimulation intensities may be unaffected by training. The cSP is hypothesized to result from gamma-aminobutyric acid (GABA-_B_)-mediated inhibition, with the initial 50 ms of this period attributed to cortical inhibition, followed by subsequent spinal inhibition (Chen et al 1999). However, recent studies have questioned whether the silent period is exclusively governed by cortical processes (Gomez-Guerrero et al 2024; Yacyshyn et al 2016). One study suggests that the first 150 ms may be explained by subcortical or spinal inhibition (Yacyshyn et al 2016). Therefore, the decreased cSP observed following both SP-RT and MP-RT in this study may be modulated at the cortical or subcortical levels or both.

### Cortico-reticular and reticulospinal changes

The present study provides novel evidence demonstrating the differential effects of RT tempo on descending motor pathways. Specifically, this is the first investigation to examine how a single session of RT modulates both cortico-reticular and reticulospinal excitability. Our findings revealed that SP-RT produced selective changes in cortico-reticulospinal excitability, as quantified by a significant increase in the index of cortico-reticulospinal activation (i.e., ICAR) compared to MP-RT.

The observed increase in ICAR following SP-RT warrants closer examination of the underlying neurophysiological mechanisms. ICAR, defined as the mathematical relationship between iMEPs and cMEPs, serves as a sensitive biomarker of the relative contributions of descending motor pathways to muscle activation (Maitland and Baker 2021). An increase in ICAR indicates enhanced reticulospinal control of the motor neuron pool, whereas a decrease suggests greater corticospinal involvement. Thus, our finding of elevated ICAR following SP-RT points to a preferential upregulation of the cortico-RST, potentially reflecting altered synaptic efficiency within the reticular nuclei and associated descending pathways.

While SP-RT appeared to induce greater modulation of responses associated with reticulospinal pathways, the differential effects observed between SP-RT and MP-RT may also reflect increased cortical engagement during MP-RT. The externally paced nature of MP-RT likely enhances reliance on cortical mechanisms, thereby attenuating the relative contribution of brainstem circuits to movement execution. This interpretation is consistent with evidence indicating that externally guided motor tasks shift the balance of motor control toward increased cortical processing (Debaere et al 2003).

The acute effects of SP-RT and MP-RT on RST efficacy were evaluated through the measure of RFD across two intervals, the first 50 ms and the subsequent 50–100 ms, under three sound conditions: visual stimuli, non-startling stimuli, and loud sound. Notably, during the Startling stimuli, RFD in the first 50 ms and subsequent 50 to 100 ms showed a significant increase at 30 min post-SP training, an effect not observed with MP-RT. Consistent with our findings, prior research suggests that fast eccentric RT, which bears similarities to SP-RT, is more effective at improving rapid force production compared to slower, more controlled movement patterns (Stasinaki et al 2019). Moreover, evidence suggests that increased RFD is associated with enhanced RST input and motor unit firing rate (Škarabot et al 2022).

The increased RFD observed following SP-RT may stem from training-induced activation of pontomedullary reticular formation neurons, leading to an elevated firing rate of α-motoneurons and, consequently, enhanced RFD. The pontomedullary reticular formation, whose function may be improved by SP-RT, is stimulated by startling stimuli, resulting in increased excitability of spinal motor neurons and subsequent improved recruitment of motor units (Del Vecchio et al 2019; Dideriksen et al 2020). The RST exerts a potent neuromodulatory influence on spinal motor neurons through the regulation of persistent inward currents, effectively modifying their activation thresholds and intrinsic excitability properties (Heckman et al 2009). A distinguishing characteristic of the reticulospinal system is its bilateral projection pattern and capacity to facilitate coordinated activation of multiple synergistic muscle groups, which enables the generation of rapid, forceful contractions that contribute substantially to improvements in RFD (Peterson et al 1975). Moreover, the enhanced RFD observed in the SP-RT group appears to be mediated by two complementary mechanisms. First, the greater total training load volume likely induced specific neural changes that optimize rapid force production capabilities. Second, high-load RT protocols have been demonstrated to augment neural drive to the motor neuron pool, facilitate more efficient motor unit recruitment patterns, and enhance the temporal precision of muscle activation through improved motor unit activation, adaptations that collectively serve to enhance the capacity for rapid force generation (Aagaard et al 2002).

Finally, it is important to acknowledge that the present study exclusively employed high-load RT protocols; therefore, the potential neurophysiological effects of light-load training were not examined. The literature demonstrates considerable variability regarding the role of contraction intensity in modulating CSE and SICI. For instance, the intensity of muscle contractions during acute bouts of RT has been shown to influence cortical excitability (Colomer-Poveda et al 2019). Several studies have reported that high-intensity strength training is required to elicit significant increases in CSE (Colomer-Poveda et al 2020; Alibazi et al 2022), and a reduction in SICI (Alibazi et al 2022; Mason et al 2019a), suggesting an intensity threshold may be necessary to drive meaningful neurophysiological adaptations. Conversely, Mason et al. (2019a) found that both heavy- and light-load strength training can acutely enhance CSE; however, light-load training did not produce measurable changes in SICI (Mason et al 2019a). These discrepancies may reflect differences in the muscle groups studied (e.g., upper vs. lower limb), their functional roles, contraction type (isometric vs. dynamic), or specific characteristics of the training protocols (e.g., SP vs. externally paced).

Training volume also appears to modulate cortical excitability, with evidence suggesting that low-intensity or light-load strength training, particularly when performed at high volumes, can enhance CSE (Colomer-Poveda et al 2019; Painter et al 2020). While the influence of contraction intensity on cortical and corticospinal adaptations is increasingly well documented, its effects on cortico-reticulospinal and reticulospinal pathway excitability remain poorly understood.

The current findings, based on high-force MP and SP contractions, highlight the need for future studies to determine whether similar neurophysiological adaptations can be elicited using lower-force contractions. Inclusion of a low-force control group in future investigations would further clarify the differential impact of contraction intensity on these neural pathways.

Understanding the functional distinctions between motor pathways is key for elucidating how different RT protocols induce specific neural adaptations. The CST and RST serve complementary roles in motor control, particularly in force production. The CST facilitates fine motor control through direct, monosynaptic connections to motor neurons innervating distal limb muscles (Lemon 2008). In contrast, the RST governs gross motor function through polysynaptic connections primarily targeting proximal and axial muscles, enabling coordinated synergistic muscle actions essential for force generation (Baker 2011). These anatomical and functional differences may explain the distinct neural responses observed between MP-RT and SP-RT. MP training, emphasizing controlled rhythmic movements, predominantly recruits the CST. Conversely, SP training, characterized by explosive gross motor actions, preferentially engages the RST. This dissociation is supported by functional neuroimaging evidence showing that paced movements activate specific cortical regions, while SP movements engage broader cortical and subcortical areas (Ackerley et al 2011). These findings suggest that MP-RT may induce adaptations primarily within the motor cortex and CST, whereas SP-RT may preferentially modify subcortical pathways, including the cortico-reticular and RSTs. Consequently, MP-RT may be more beneficial for tasks requiring precise timing, while SP-RT might optimize gross motor performance and overall strength development.

The distinct neurophysiological responses elicited by SP-RT and MP-RT have important implications for long-term neuromuscular performance and rehabilitation strategies. SP-RT preferentially enhances RST excitability, which is particularly relevant for populations requiring rapid force production and postural stability, such as older adults at risk of falls or individuals with neuromuscular impairments affecting balance and mobility (Atkinson et al 2022; Baker 2011). Given the RST’s key role in facilitating proximal muscle activation and rapid force execution, repeated exposure to SP-RT may drive plastic adaptations that enhance RFD, dynamic stability, and functional independence in aging populations (Aagaard et al 2010; Clark & Fielding 2012).

The RST is increasingly recognized as a critical compensatory motor pathway in individuals with CST damage, including those with spinal cord injury or post-stroke cortical lesions (Baker & Perez 2017; Zaaimi et al 2012). As SP-RT may promote RST excitability, it holds potential as a therapeutic intervention to augment motor recovery in individuals with limited CST function, possibly facilitating functional restoration through reticulospinal plasticity (Baker & Perez 2017; Darling et al 2011). In contrast, MP-RT primarily enhances cortical and corticospinal excitability, which may benefit individuals requiring precise voluntary motor control, such as stroke survivors with partial CST preservation or individuals with neurodegenerative conditions affecting fine motor coordination (Colomer-Poveda et al 2021; David et al 2012; Gordon et al 2024).

### Limitations

This study is the first to investigate the acute effects of different types of RT on cortico-reticular and RST excitability, highlighting distinct changes in cortical, corticospinal, corticoreticular, and RST activity following varied RT approaches. Despite these novel contributions, some limitations warrant consideration to accurately contextualize the findings.

Efforts were made to control for factors such as age, sex, time of day (to address diurnal training effects), handedness, and menstrual cycle phase. However, potential influences from factors beyond the study’s scope, such as mechanical, metabolic, and hormonal responses, could still impact our findings. Additionally, our results are generalizable only to a population of healthy, young, novice participants and may not apply to other groups, such as older adults or athletes with varying training backgrounds. Moreover, this study included a relatively small sample size in each group (*n* = 12), which may limit the generalizability of the findings. While the study was adequately powered to detect within-group and between-group differences, the sample size may not fully account for inter-individual variability, particularly given the sex distribution (27 males, 9 females). Future studies with larger and more balanced cohorts are needed to confirm these findings and explore potential sex-specific differences in training-induced neurophysiological adaptations.

There may also be some reporting bias regarding participants’ sedentary status, as outcomes could differ if individuals with higher physical activity levels were included. Moreover, although all participants received standardized instructions and consistent verbal encouragement to ensure maximal effort during testing, we acknowledge that motivation could still have influenced the observed acute effects and should be considered when interpreting the results. Another potential limitation is the possibility that repeated testing may have influenced participant fatigue and recovery. While no significant decreases in M_MAX_ or MVF were observed and rest periods were provided between tests, the cumulative effects of multiple testings cannot be entirely excluded. Future studies should consider incorporating additional measures to monitor fatigue and recovery or refining testing protocols to mitigate these potential effects.

Another limitation pertains to the assessment of RST excitability using indirect measures, RFD. Though it provides valuable insight, it does not allow for direct measurement of RST activity. Similarly, while TMS provides valuable indices of cortico-reticulospinal excitability through measurement of iMEP, it does not directly examine RST contributions. Therefore, our findings should be interpreted with these study limitations in mind.

## Conclusions

The findings demonstrate distinct acute effects of MP-RT and SP-RT on neurophysiological measures. MP-RT significantly increased corticospinal excitability and reduced cortical inhibition, while SP-RT primarily enhanced cortico-reticular and reticulospinal excitability at 30 min post-RT. This indicates that MP-RT enhances cortical excitability, whereas SP-RT strengthens cortico-reticular and reticulospinal excitability. These immediate, short-term responses following a single training session may serve as an indicator of long-term neuroplastic adaptations. Investigating the long-term effects of MP-RT and SP-RT on corticoreticular and reticulospinal pathways is essential to determine whether acute neurophysiological responses persist and contribute to sustained strength gains. Additionally, examining MP training modalities in populations with neurological impairments, such as stroke survivors or individuals with spinal cord injury, could clarify their potential for enhancing motor recovery and functional rehabilitation.

### Perspective

This study elucidates the distinct neurophysiological responses resulting from MP-RT and SP-RT, providing valuable insights into their targeted applications. MP-RT significantly enhances corticospinal excitability and intracortical facilitation, suggesting its potential utility for activities requiring precise motor control, such as skill-based sports or the rehabilitation of fine motor deficits. In contrast, SP-RT facilitates improvements in cortico-reticular and reticulospinal excitability, thereby promoting more rapid and robust motor outputs. This may be particularly advantageous for explosive tasks, including sprinting, powerlifting, or functional movements in daily life.

Overall, these findings underscore the importance of tailoring RT modalities to specific neuromuscular objectives, thereby informing evidence-based exercise prescriptions in athletic training, physical therapy, and motor recovery programs. Future studies should investigate whether the hypothesized training adaptations correspond with the distinct neurophysiological targets and functional outcomes of each modality; specifically, whether MP-RT engages the CST to enhance fine motor control, and SP-RT engages the RST to more effectively improve gross motor function and strength in healthy, untrained adults over weeks or months of RT. Upon confirmation in healthy populations, it will be essential to extend these investigations to clinical groups with neurological impairments, such as stroke or spinal cord injury, to further elucidate their role in promoting motor recovery and rehabilitation outcomes.

## Supplementary Information

Below is the link to the electronic supplementary material.

Supplementary file1 Supplementary Figure 1. Raw sEMG traces showing the Motor Evoked Potential (MEP) and Cortical Silent Period (cSP) of the biceps brachii muscle from a single participant in each group: (A) Control, (B) Self-paced (SP), and (C) Metronome-paced (MP). Traces are shown at Baseline, Post 5 minutes, and Post 30 minutes following resistance training (RT). A decrease in cSP duration at Post 5 and Post 30 for the MP and SP groups is indicated by green color arrows. (JPEG 30 KB)

Supplementary file2 Supplementary Figure 2(a, b and C). Raw sEMG traces showing the cMEP and iMEP of the biceps brachii muscle from a single participant in each group: (A) Control, (B) Metronome-paced, and (C) Self-paced resistance training group. Traces are displayed at Baseline, Post 5 minutes, and Post 30 minutes following resistance training (RT). The iMEP is observed to occur more than 5 ms later than the cMEP (black arrows indicate cMEP onset, while the green arrows indicate iMEP onset), confirming that it is a true iMEP. cMEP: contralateral Motor Evoked Potential; iMEP: Ipsilateral Motor Evoked Potential. (JPEG 20 KB)

Supplementary file3 (JPEG 21 KB)

Supplementary file4 (JPEG 18 KB)

## Data Availability

Data for the experiments reported here can be made available upon reasonable request.

## Abbreviations

AMT: Active motor threshold
AURC: Area under the recruitment curve
CST: Corticospinal tract
cSP: Cortical silent period
EMG: Electromyography
EMGrms: EMG root-mean-square
ICAR: Ipsilateral to contralateral motor evoked potential amplitude ratio
ICF: Intracortical facilitation
MEP: Motor evoked potential
M_MAX_: Maximal compound action potential
MVF: Maximal voluntary force
MP-RT: Metronome-paced resistance training
SP-RT: Self-paced resistance training
RFD: Rate of force development
RM: Repetition maximum
RT: Resistance training
RST: Reticulospinal tract
sEMG: Surface electromyography
SICI: Short-interval cortical inhibition
TMS: Transcranial magnetic stimulation
