# Supplementary figures and images for: Determining the cortical, corticospinal, and reticulospinal responses to metronome-paced and self-paced strength training
Source: Eur J Appl Physiol. 2025 Aug 31;126(3):1333–56. doi: 10.1007/s00421-025-05939-3 (PMC13013172; doi:10.1007/s00421-025-05939-3)

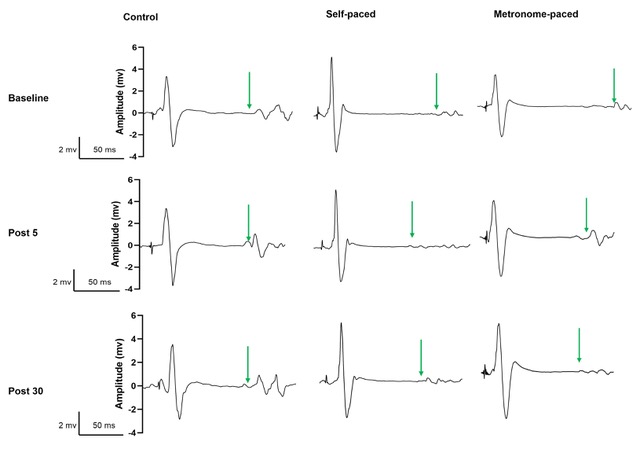

Supplement: Supplementary file 1 — Supplementary file1 Supplementary Figure 1. Raw sEMG traces showing the Motor Evoked Potential (MEP) and Cortical Silent Period (cSP) of the biceps brachii muscle from a single participant in each group: (A) Control, (B) Self-paced (SP), and (C) Metronome-paced (MP). Traces are shown at Baseline, Post 5 minutes, and Post 30 minutes following resistance training (RT). A decrease in cSP duration at Post 5 and Post 30 for the MP and SP groups is indicated by green color arrows. (JPEG 30 KB) [file 421_2025_5939_MOESM1_ESM.jpeg]

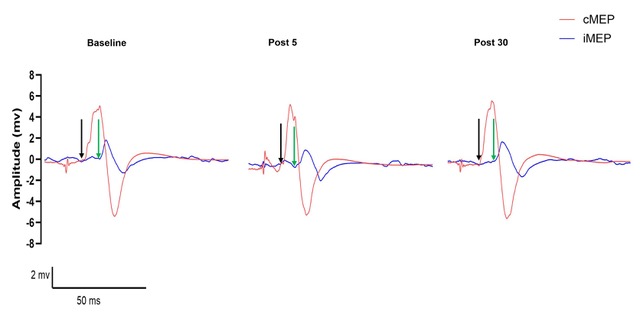

Supplement: Supplementary file 2 — Supplementary file2 Supplementary Figure 2(a, b and C). Raw sEMG traces showing the cMEP and iMEP of the biceps brachii muscle from a single participant in each group: (A) Control, (B) Metronome-paced, and (C) Self-paced resistance training group. Traces are displayed at Baseline, Post 5 minutes, and Post 30 minutes following resistance training (RT). The iMEP is observed to occur more than 5 ms later than the cMEP (black arrows indicate cMEP onset, while the green arrows indicate iMEP onset), confirming that it is a true iMEP. cMEP: contralateral Motor Evoked Potential; iMEP: Ipsilateral Motor Evoked Potential. (JPEG 20 KB) [file 421_2025_5939_MOESM2_ESM.jpeg]

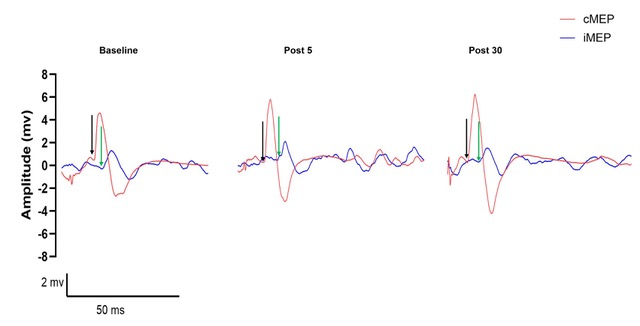

Supplement: Supplementary file 3 — Supplementary file3 (JPEG 21 KB) [file 421_2025_5939_MOESM3_ESM.jpeg]

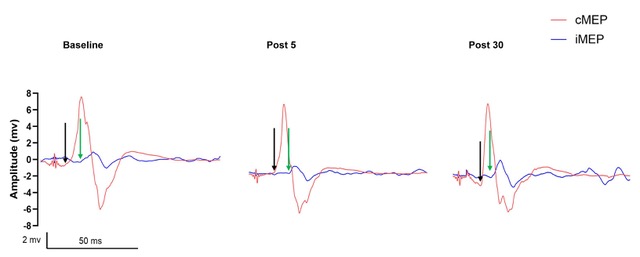

Supplement: Supplementary file 4 — Supplementary file4 (JPEG 18 KB) [file 421_2025_5939_MOESM4_ESM.jpeg]
